# Neuroelectric Tuning of Cortical Oscillations by Apical Dendrites in Loop Circuits

**DOI:** 10.3389/fnsys.2017.00037

**Published:** 2017-06-14

**Authors:** David LaBerge, Ray S. Kasevich

**Affiliations:** ^1^Department of Cognitive Sciences, University of California, Irvine, IrvineCA, United States; ^2^Stanley Laboratory of Electrical Physics, Great BarringtonMA, United States; ^3^Bard College at Simon’s Rock, Great BarringtonMA, United States

**Keywords:** oscillations, apical dendrite, pyramidal neuron, thalamus, neural clock, loop circuits, network circuits

## Abstract

Bundles of relatively long apical dendrites dominate the neurons that make up the thickness of the cerebral cortex. It is proposed that a major function of the apical dendrite is to produce sustained oscillations at a specific frequency that can serve as a common timing unit for the processing of information in circuits connected to that apical dendrite. Many layer 5 and 6 pyramidal neurons are connected to thalamic neurons in loop circuits. A model of the apical dendrites of these pyramidal neurons has been used to simulate the electric activity of the apical dendrite. The results of that simulation demonstrated that subthreshold electric pulses in these apical dendrites can be tuned to specific frequencies and also can be fine-tuned to narrow bandwidths of less than one Hertz (1 Hz). Synchronous pulse outputs from the circuit loops containing apical dendrites can tune subthreshold membrane oscillations of neurons they contact. When the pulse outputs are finely tuned, they function as a local “clock,” which enables the contacted neurons to synchronously communicate with each other. Thus, a shared tuning frequency can select neurons for membership in a circuit. Unlike layer 6 apical dendrites, layer 5 apical dendrites can produce burst firing in many of their neurons, which increases the amplitude of signals in the neurons they contact. This difference in amplitude of signals serves as basis of selecting a sub-circuit for specialized processing (e.g., sustained attention) within the typically larger layer 6-based circuit. After examining the sustaining of oscillations in loop circuits and the processing of spikes in network circuits, we propose that cortical functioning can be globally viewed as two systems: a loop system and a network system. The loop system oscillations influence the network system’s timing and amplitude of pulse signals, both of which can select circuits that are momentarily dominant in cortical activity.

## Introduction

The most numerous type of neuron in the cerebral cortex is the pyramidal neuron, which forms 70–80% of the neurons in the mammalian cortex (Feldman, 1984). Its distinguishing features are a pyramid-shaped soma and a relatively long dendrite extending upward from the apex of the soma. Owing to its vertical orientation (i.e., being perpendicular to the cortical surface) and its considerable length, the apical dendrite can easily be observed in micro-photographs among the several shorter basal dendrites which leave the soma in many other orientations. Although the considerable length of the apical dendrite makes it stand out when one observes networks of cortical neurons, its length apparently contributes no special function that stands out when we try to explain how these networks of neurons work. Thus, in the conventional view the apical dendrite’s most distinct anatomical feature does not correspond to a distinct functional feature.

Specifically, it is not clear to contemporary neuroscientists that the apical dendrite does anything for cortical processing that is not also done by the several shorter basal dendrites which arise from the soma of the pyramidal neuron. The prevailing view of the main function of the apical and basal dendrites of a pyramidal neuron is the integration of incoming electric pulses at the tens of thousands of synapses which dot the dendrite surface (e.g., Häusser et al., 2000; Spruston, 2008) The result of this integration is a train of output pulses in the single axon which exits at the base of the soma.

The present study is concerned with the influence of apical dendrite activity on information processing in cortical networks. Here, we propose that a major function of the long apical dendrite in pyramidal neurons is the production of a stable oscillation at a specific frequency. When apical dendrites in corticothalamic loops oscillate, their oscillations are copied to networks of pyramidal neurons, and all network pyramidal neurons that are contacted will oscillate at a common carrier frequency into which messages of temporally coded spikes may be inserted. Only neurons whose membranes oscillate at the specific carrier frequency will accept the spike signals: spike signals carried on other frequencies will be blocked.

The synchronous rhythms of oscillations within cortical circuits are currently being investigated in neuroscience at several fronts with different techniques. A pervasive paradox is the apparently high levels of randomness in spike discharge and the apparently high levels of orderliness in oscillations. A thorough review of relevant experiments and theories by Wang (2010) provides a window onto a bewildering scene of data and models about how circuits, neurons, and membrane conductances can together or separately produce oscillatory activity. Of particular relevance to the present study are oscillations of the neuronal membrane at levels subthreshold to action potentials, and the tendency of an apical or basal dendritic membrane to oscillate at a particular peak resonant frequency.

We use the term *tuning* to refer to operations that produce a particular frequency of oscillation in an apical dendrite. *Fine-tuning* produces a significant reduction in the frequency bandwidth and noise levels. This refinement of the frequency bandwidth is necessary in order to maintain a virtually constant duration of each frequency cycle within the interconnected circuit.

The corticothalamic loops operate as a clock in a computer, which assures that electric signals in different locations within a connected circuit of neurons change at the same time. In the present theory, different bundles of apical dendrites can oscillate at different frequencies, and therefore their connected circuits of neurons can run on different clocks.

Adjustment of the temporal unit of the apical dendrite “clock” is based on producing a steady train of subthreshold pulses along the dendritic shaft of the pyramidal neuron. The durations of intervals between pulses approaches a constant value as frequencies reduce their variability toward zero. Reduction of frequency variability therefore is one of the two central operations of the present model of apical dendrite neuronal tuning. The other central operation of the present model is the setting of the modal frequency of oscillation, also termed the resonant frequency of the oscillation.

Thus, the apical dendrite clock serves to coordinate the timing of spike signals throughout the group of neurons having synaptic contact with the pyramidal neuron that contains the apical dendrite. Spike signals are typically of very short duration, and the maintaining of high precision in the temporal coordination of short-duration signals enables them to be effectively integrated when they converge at sites on dendrites and somas. Without fine-tuning of temporal intervals in wires and fibers that carry information, computation as we know it today would not be possible.

## The Long Apical Dendrites of Cortical Layers 5 And 6 Pyramidal Neurons

**FIGURE 1:**
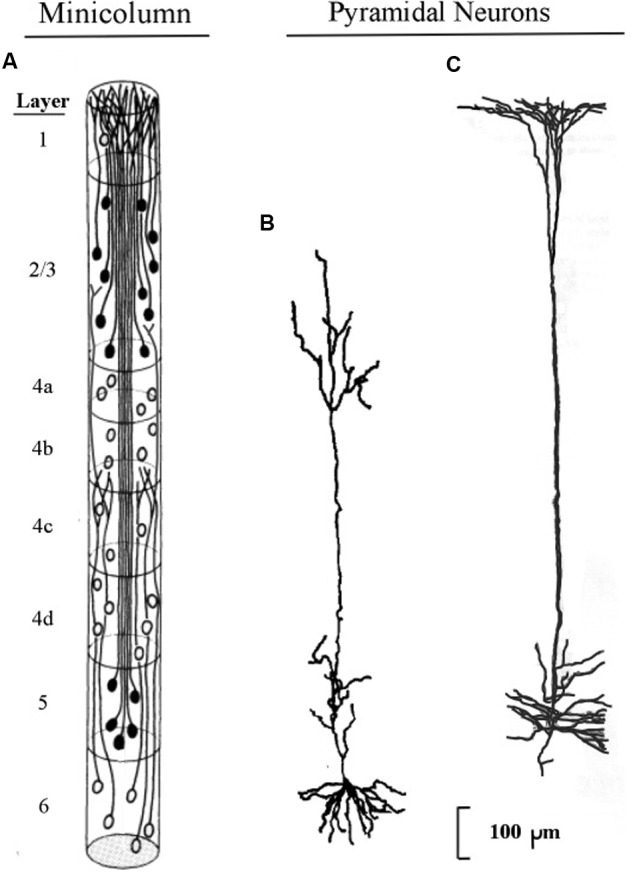
**Pyramidal neurons with long apical dendrites from layers 5 and 6. (A)** Minicolumn diagram adapted from Peters and Sethares (1991) of a slice of monkey visual cortex, which shows locations of somas and apical dendrites (basal dendrites and axons are omitted). Somas showing no attached fibers are stellate neurons, which contain many short dendrites that radiate from the soma in all directions (not shown here). The vertical length of the minicolumn indicates the thickness of the cortical area in which the two pyramids were observed. **(B)** Camera lucida drawing of a layer 6 pyramidal neuron of the monkey from Figure 4 of Wiser and Callaway (1996); **(C)** Camera lucida drawing of a layer 5 pyramidal neuron of the monkey from Figure 24 of Valverde (1986).

The longest apical dendrites in the cerebral cortex arise from the apex of pyramidal neurons (pyramids) whose somas lie in layer 5 or layer 6. **Figure 1** shows the examples of pyramidal neurons taken from each of these cortical layers. On the left side of **Figure 1**, the pyramidal neurons are depicted diagrammatically as oval shapes, and the relatively long line attached at the top of an oval shape represents the apical dendrite. Thus, the axon and the many basal dendrites of the pyramids are omitted in the minicolumn diagram.

In **Figure 1**, the filled oval shapes located in layers 2, 3, and 5 represent pyramidal somas whose apical dendrites cluster together to form the center of a minicolumn, according to Peters (1994). The unfilled oval shapes located in layer 6 represent pyramidal somas whose apical dendrites join with layer 6 apical dendrites of 80–100 other minicolumns to form the interior area of a column. The minicolumn structure shown in **Figure 1** serves as a convenient framework for positioning neurons in circuit diagrams, while we postpone to the “Discussion” Section an examination of how the current concepts of minicolumn, column, and apical dendrite bundle correspond to a functional unit of cortical action.

Visual inspection of **Figure 1** gives the impression that the considerable majority of neurons in the cerebral cortex are pyramidal neurons, which is confirmed by Feldman’s (1984) measurements, which estimate their percentage in the cortex as approximately 70–80%. The remaining percentages of stellate and inhibitory neurons (in area V1) are approximately 5–8% and 16–20%, respectively (Mountcastle, 1998). Under the area of a dime placed on the human skull over the parietal cortex there are approximately 18,000,000–20,000,000 pyramidal neurons, (based on a cell count of approximately 70 neurons beneath surface patches of 25 × 30 microns for the monkey).

When clusters of minicolumns are viewed in magnification, the apical dendrites from layers 5 and 6 give a slice of cortex its visual resemblance to a grove of tall, tightly packed tree trunks. Among mammals, layer 5 apical dendrites in the parietal area increase in length from mouse to human by a factor of approximately 4.2, while the thickness of the parietal cortex varies by a factor of approximately 4.0 (see Figure 2 of Rockel et al., 1980). Hence, the increase of cortical thickness from mouse to human appears to correlate closely with the length of the apical dendrites arising from layer 5 pyramids.

The structure of the minicolumn is organized around the central core of layer 5 pyramids (Peters and Sethares, 1991). Layer 6 pyramids are not organized within the minicolumn but instead are organized within the column, which is a bundle of approximately 100 minicolumns (Mountcastle, 1998). The minicolumn is regarded as the functional unit of the cortex (Mountcastle, 1957), based on the observation that neurons in a given minicolumn share many receptive field properties.

The axons of pyramidal neurons of layers 2 and 3 form an information bridge from one minicolumn to other minicolumns at locations both near and far. Axons of layer 5 pyramids contact minicolumns near and far, so that oscillations of the long apical dendrites positioned vertically at the center of a minicolumn can be synchronized with oscillations of neurons throughout a broad network of neurons. When membranes of network neurons oscillate in synchrony the wave peaks of these oscillations are separated by a constant time interval; so it can be said that these neurons bind together and “talk to each other” (Fries, 2005, 2015; Womelsdorf et al., 2007).

To summarize this section, the oscillations of layer 5 and 6 apical dendrites drive the output pulses of their pyramidal axons, which are copied to the thalamus and sent back to the same apical dendrite, forming a loop circuit. The thalamic axon also sends these pulses to other neurons to which it is connected, some located nearby, some located remotely. In this manner, oscillations of the same frequency as those in the initiating apical dendrites can be spread across the cortex.

## Modeling the Electric Oscillations of the Apical Dendrite

### Brief Review of a Biophysical Model of Apical Dendrite Oscillations

Two features of oscillations are prominent in the present modeling of apical dendrite activity. The first is the peak frequency of the resonance profile of oscillations, and the second is the progressive narrowing of the resonance profile around the peak frequency. When a neuron is induced to oscillate at a particular frequency, we say that it is *tuned* at its peak frequency, and when the distribution around the peak oscillation frequency is made very narrow we say that it is *fine-tuned*.

The hypothesis that as the excitatory postsynaptic potential (EPSP) (pulse surge) moves down the apical dendrite the intensity profile is made more narrow was explored and described graphically in an earlier paper (LaBerge, 2005). A detailed quantitative description of the narrowing process for frequency was subsequently presented by Kasevich and LaBerge (2011).

A basic assumption of the model is that biochemical properties of the apical dendrite membrane narrow the resonance profile of oscillations as pulse-like surges of electric current pass repeatedly down the apical dendrite. The voltages of these pulse-surges are presumed almost always to be subthreshold for the production of action potential spikes. When an occasional action potential spike does occur, it is assumed to arise from conditions external to the surges of current in the EPSP delivered by the thalamic axon, for example, back-propagation from a spike generated at the soma, or by activation of calcium channels.

**FIGURE 2:**
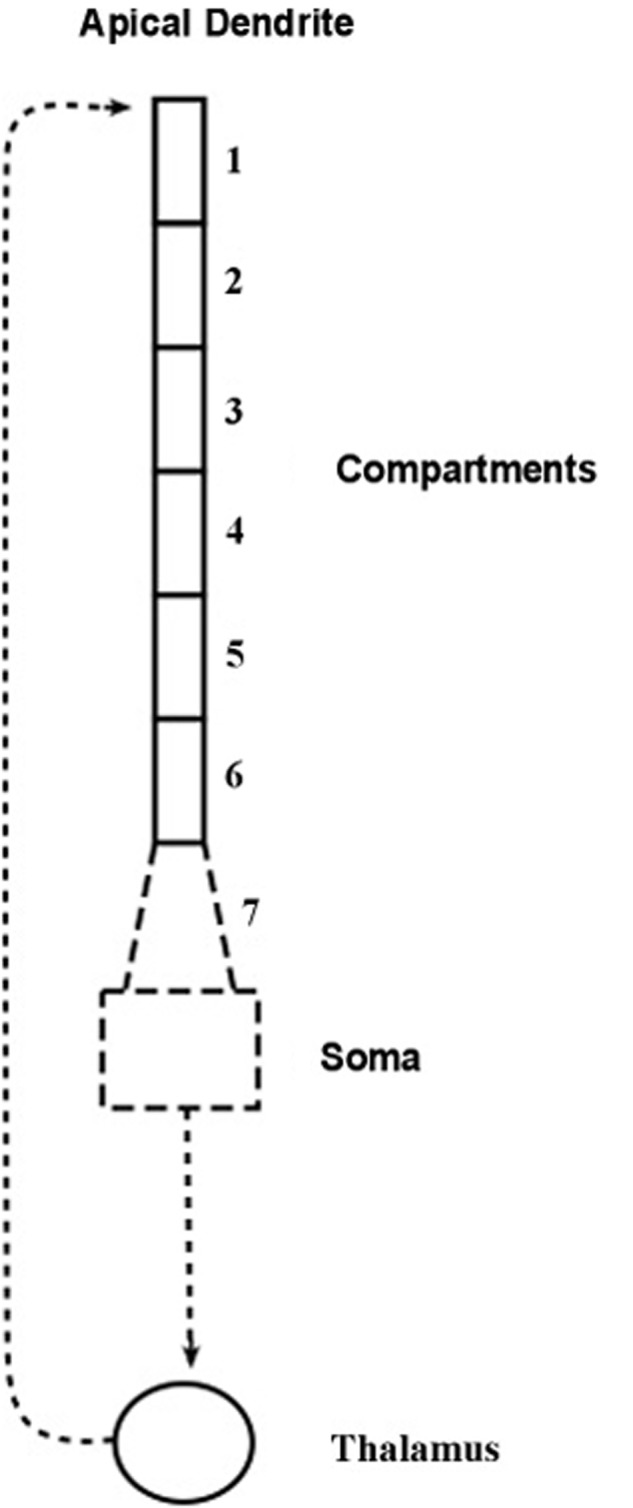
**Compartment model of the apical dendrite**. Six compartments correspond to the lengthy mid-range of the apical dendrite. An additional seventh compartment corresponds to the transitional segment located between the soma and the mid-range of the apical dendrite. The width of the dendrite tapers from the large soma and adjacent locations of basal dendrite attachments, and as it tapers it also loses inhibitory synapses and begins gradually to add spines containing excitatory synapses. [Adapted from Figure 1 of Kasevich and LaBerge (2011)].

The compartmental model, shown diagrammatically in **Figure 2**, describes how electrical energy is passed down the apical dendrite of a pyramidal neuron to the soma, starting from synaptic inputs located at the top of the dendrite. The pyramidal axon connects with a thalamic neuron whose axon returns to synapse at the top of the apical dendrite of origin.

The structure of the present model follows the compartmental reconstruction of the apical dendrite by Destexhe (2001). Here, **Figure 2** shows six compartments representing the relatively long mid-region of the apical dendrite, while the seventh compartment is proximal to the soma. The diameter of this transitional compartment gradually decreases to accommodate the large difference in diameters of the soma and the mid-region of the dendrite. The remaining six compartments maintain a relatively constant diameter (Keren et al., 2005). The synapses of the initial 40 μm part of the proximal seventh compartment, like the soma, are inhibitory and excitatory spines are absent (Valverde, 1967; DeFelipe and Fariñas, 1992). At the distance from the soma where the six remaining compartments begin, the synapses are mostly excitatory (Keren et al., 2009). In view of these considerations it is presumed that the contribution to frequency narrowing by the transitional compartment can be ignored in the present treatment of the model.

**FIGURE 3:**
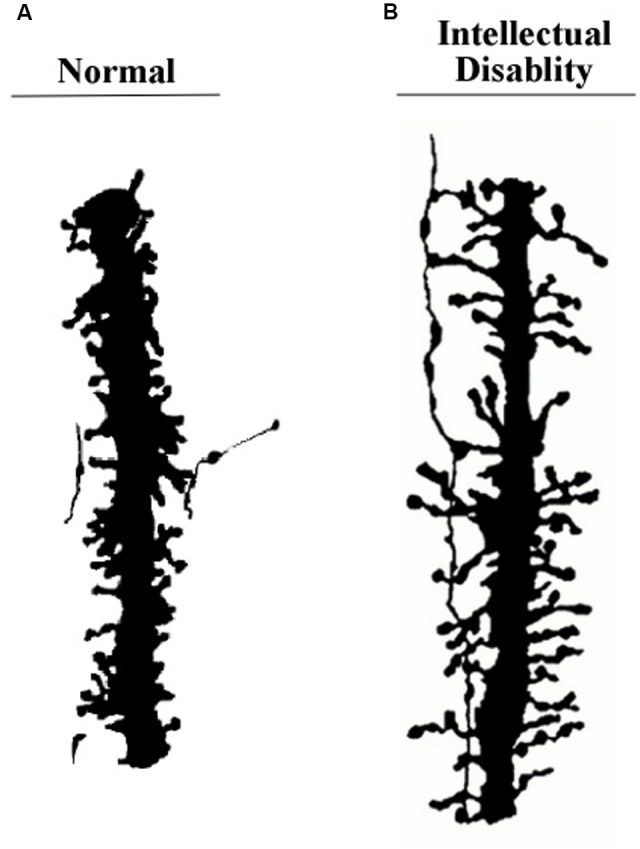
**Segments of apical dendrites of the newborn human infants. (A)** Normal; **(B)** intellectual disability probably related to abnormally long spines. Remnants from an axon contacting the spines suggests a general vertical orientation consistent with the axons shown in **Figure 10B**. Adapted from Marin-Padilla (1972).

One of the most important features of apical dendrites (and dendrites in general) are the thousands of spines that crowd their surfaces. **Figure 3** shows two examples of segments of apical dendrites taken of newborn children, one normal, and the other with symptoms of an intellectual disability.

Long vertically oriented axons, similar to the apical dendrite segment shown on the right side of **Figure 3**, have been found running along apical dendrites of layer 6 pyramidal neurons. An example from Sherman and Guillery (2011) is shown in **Figure 10**.

**FIGURE 4:**
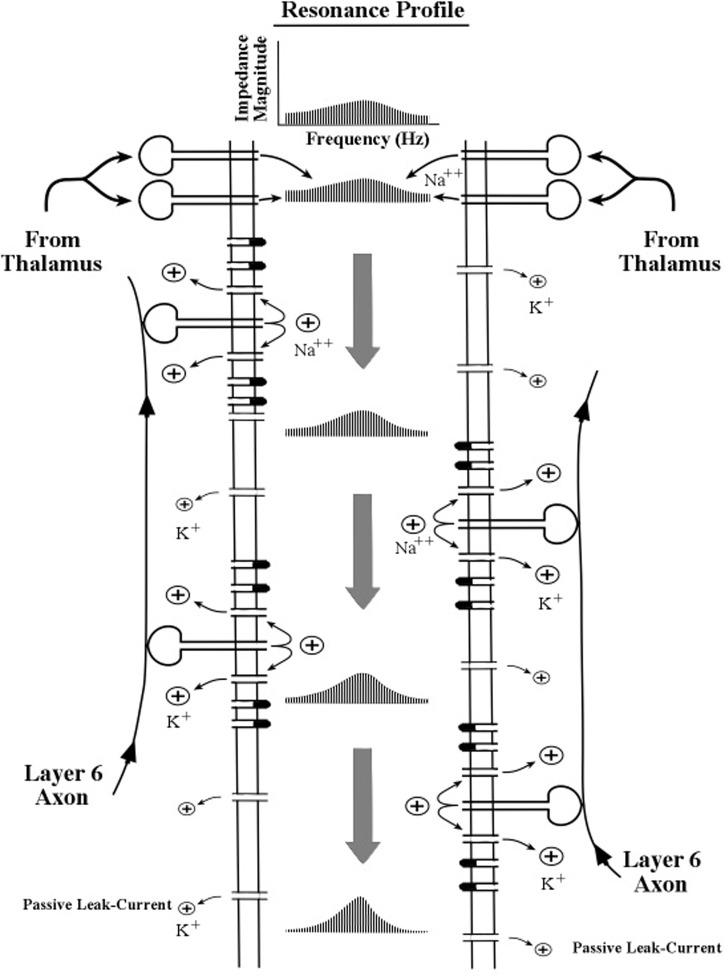
**Schematic diagram of the interior of an apical dendrite located near the sites of the thalamocortical axon input**. The border of the interior region is formed by a thin membrane containing a high density of channels (gates) including the inward sodium (Na++) channels in the spines and the outward potassium (K+) channels in the membrane. The flow of ion charges through these channels operates on the surges of current as they pass down the apical dendrite to shape the resonance profile of push–pause oscillations. Other membrane channels are omitted for clarity. The description for the narrowing of the profile is given by Equation [19] in Kasevich and LaBerge (2011).

To aid in the description of the biophysical operations within the apical dendrite we use a schematic diagram of apical dendrite segment in **Figure 4**. Oversized drawings of spines can be seen piercing the apical dendrite membrane. The flow of ion charges through these channels operates on the surges of current as they pass down the apical dendrite to shape the resonance profile of push–pause oscillations. Other membrane channels are omitted for clarity. The description for the narrowing of the profile is given by Equation [19] in Kasevich and LaBerge (2011).

The modeling of oscillations at any given location along the apical dendrite is represented theoretically by a distribution of resonance frequencies, which is visually depicted as a resonance profile. Two features of the profile are evident and of main interest: its peak and its spread. The peak frequency of the resonance profile is customarily called the resonant frequency. As it is most generally used, the term resonance denotes the ability of a system to oscillate most strongly at a particular frequency. It may be noted that the existence of resonance in this biophysical model does not require inductance, in contrast with most classical electrical circuitry that is tuned to a specific frequency.

Both the peak resonance frequency and the progressive narrowing process are assumed to be influenced by many features of the membrane, but chief among these features is the inward flow of positive sodium ions by spines, the consequent outward flow of positive potassium ions around the spines, the passive outflow of potassium ions by the leak current, and to a lesser extent the activities of many other types of channels in the dendritic membrane.

The initial surge of charge consists of action potentials which are delivered by a thalamic axon at the top of the apical dendrite. The quantity of positive ions in a single EPSP (excitatory postsynaptic potential) can vary according to the number of momentarily contributing synapses (spatial summation) and/or by the number of pulses delivered to a synapse in a very short time period (temporal summation), as from a burst of action potentials. This variable level of voltage amplitude of a current surge in the apical dendrite contrasts with the discrete, all-or-none voltage amplitude of an action potential spike propagating along an axon of the neuron.

As successive EPSPs pass along the shaft of the apical dendrite (shown in **Figure 4**), the thin membrane located on the walls of the apical dendrite oscillates electrically with these successive surges of current flowing down the center of the dendritic shaft. Owing to the biophysical features of the membrane, the intervals between surges of current may be modified slightly as they move down the dendrite. As the interval between pulse-surges changes so does the momentary frequency of oscillation. Longer intervals correspond to lower frequencies and shorter intervals correspond to higher frequencies.

### Local Control of the Peak Frequency of Apical Dendrite Oscillations by Membrane Channel Activities

As current surges flow down the apical dendrite their oscillation frequency is assumed to change slightly when local potassium channels change their rate of releasing potassium ions from the dendrite. According to the equations given in Kasevich and LaBerge (2011) the rate of outward flow through a particular potassium channel is regulated by the momentary level of voltage on the inside surface of the membrane surrounding the channel. This level of internal voltage is maintained by the synaptic discharges from local spines, which inject positive sodium ions onto the internal surface of the dendrite. The synaptic discharges in the spines, in turn, are triggered by trains of electric pulses from local axons that contact the spines.

**FIGURE 5:**
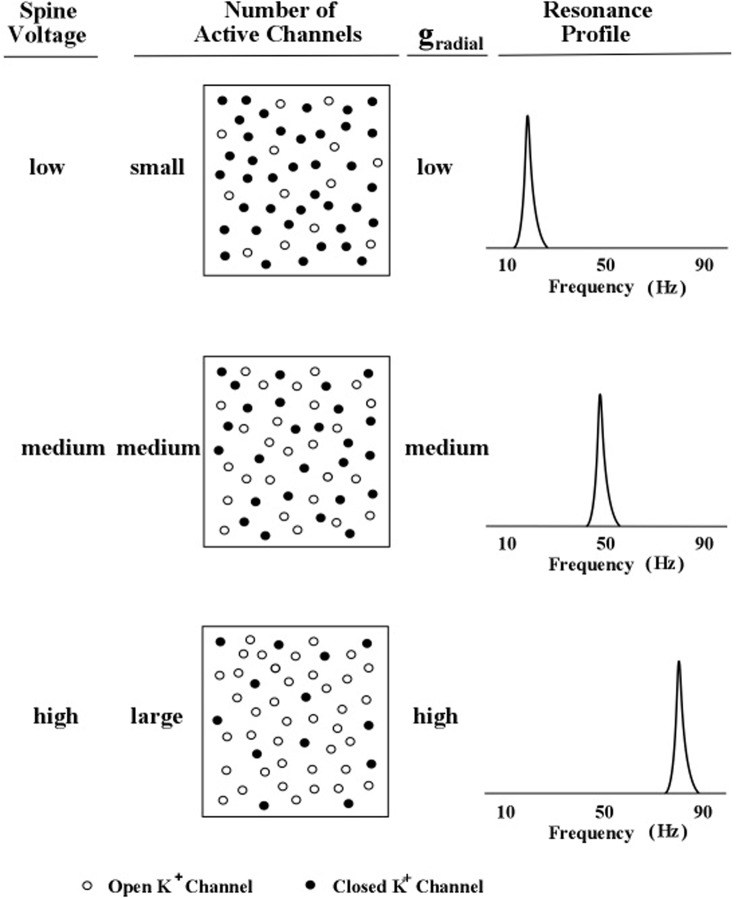
**Profile peak frequency and activity of local spines**. A schematic diagram of the hypothesized relationship between the peak frequency of a profile curve and the internal voltage level produced by subthreshold activity of an increasing number of local dendritic spines. The rate of outward (radial) potassium flow is denoted by *g*_r_. Levels of spine voltage are low, medium, and high; number of active channels are small, medium, and large; and levels of *g*_r_ are low, medium, and high (Kasevich and LaBerge, 2011).

The relationship between spine-delivered voltage and peak frequency is graphically illustrated in **Figure 5**. Three levels of outward flow of potassium ions are shown in **Figure 5**.

In **Figure 5**, the outward flow of potassium ions is represented in the model by the parameter, *g*_r_, which denotes the value of the potassium channel radial conductance. Movement of potassium ions out of the dendrite lowers the voltage inside the dendrite, which contrasts with the movement of sodium into the dendrite (via the spine) which increases the voltage inside the dendrite. Two kinds of potassium channels are featured in the present model of apical dendrite activity: the leak current and the voltage-gated current. The leak current is represented in **Figure 5** by the smallest plus signs moving outward across the membrane, and the voltage-gated current is represented by the larger plus signs moving outward across the membrane. The rate of outward flow for the leak current is assumed to be small and constant; the rate of outward flow for the voltage-gated current depends upon the local voltage inside the membrane, which is determined by the sodium ions entering through the neighboring spines. Low spine voltage produces low outward potassium flow and low values of oscillation frequency, and high spine voltage produces high potassium flow and high values of oscillation frequency. Thus, the present model predicts that the rate of outward potassium flow through the apical dendrite membrane determines the peak frequency of the dendrite’s oscillations. Measurements of these oscillations can be obtained from the local electric field potentials that they produce; and as the field potentials of clusters (bundles) of thousands of apical dendrites radiate to the scalp their combined voltage can be measured as EEGs.

### Narrowing the Resonance Profile by Repeated Movements of Current Pulses Down the Apical Dendrite

When the corticothalamic loop shows oscillatory activity, pulse surges of current repeatedly pass along the membrane (see **Figure 4**). After each complete pass along the dendrite, the resonance profile narrows only slightly, so that many cycles of surges are needed to sharpen the profile to the point where the frequency of surges are fine-tuned close to one specific frequency.

As the pulse-surges move repeatedly down the apical dendrite the outflow of potassium ions changes the shape of the resonance profile, compartment after compartment. The narrowing of the profile comes about by increasing the occurrences of specific resonance frequencies near the peak resonance frequency region of the profile and decreasing the occurrences of specific resonance frequencies remote from the peak frequency. In Kasevich and LaBerge (2011) Equation [19] describes this change in shape.

## Results of the Simulation Experiment

### Peak Frequency as a Function of Rate of Ion Outflow

Four equally spaced frequencies in the 0–100 Hz range were selected, 20, 40, 60, and 80 Hz, and the outward flow of potassium ions, *g*_r_, was calculated for each frequency using Equation [1] of Kasevich and LaBerge (2011), which is based on a single compartment. Iterative calculations converged to a value of *g*_r_ that maximized the energy transfer from one compartment to the next compartment. Appropriate geometric parameters of the underlying leaky cable theory and electrical parameters of the membrane were obtained from the relevant literature and entered into Equation [12] of the Kasevich and LaBerge (2011) study.

**FIGURE 6:**
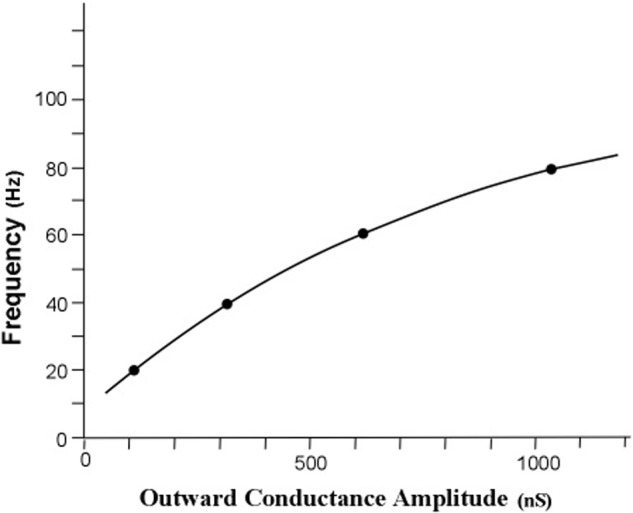
**Simulated relationship of profile peak frequency as a function of the rate of outward conductance of potassium ions**. The obtained outward potassium conductances were 105, 310, 627, and 1056 nS for the curves with peaks at 20, 40, 60, and 80 Hz, respectively. The resonant frequency in radians per second is approximately the radial conductance divided by the radial capacitance. For a constant radial capacitance the peak frequency is directly proportional to the potassium ion leak conductance. [Adapted from Kasevich and LaBerge (2011)].

The measure of energy transfer was transfer impedance. The relationship between the obtained *g*_r_ conductance and each of the four frequency points is graphed in **Figure 6**.

The first result of the simulation is that the iterative calculations of the main equation revealed an orderly relationship between peak frequency and outward potassium conductance, *g*_r_. Increasing the outward conductance increases the frequency of the peak of the frequency profile, but the peak frequency appears to approach a limit as outward conductance increased toward higher values. This result confirms the first tuning hypothesis, which is that the peak frequency of an apical dendrite oscillation can be set by adjusting the rate of outward flow of potassium ions through the apical dendrite membrane.

### Narrowing the Frequency Profile

The second main result of the simulation concerns the stability of the peak resonance frequency during repeated cycling of pulses within the corticothalamic loop circuit. Using Equation [19] in the study by Kasevich and LaBerge (2011), we simulated this cyclic activity in an attempt to discover whether or not the profile of pulse frequency revealed a progressive sharpening around a peak frequency that reached an asymptote of zero or near zero. The four peak frequencies, 20, 40, 60, and 80 Hz and their respective values of *g*_r_ (the outward voltage-gated conductance) were entered into the difference equation (Equations [16] and [19]) which describes the compartment-by-compartment change in the frequencies that make up the resonance profile.

**FIGURE 7:**
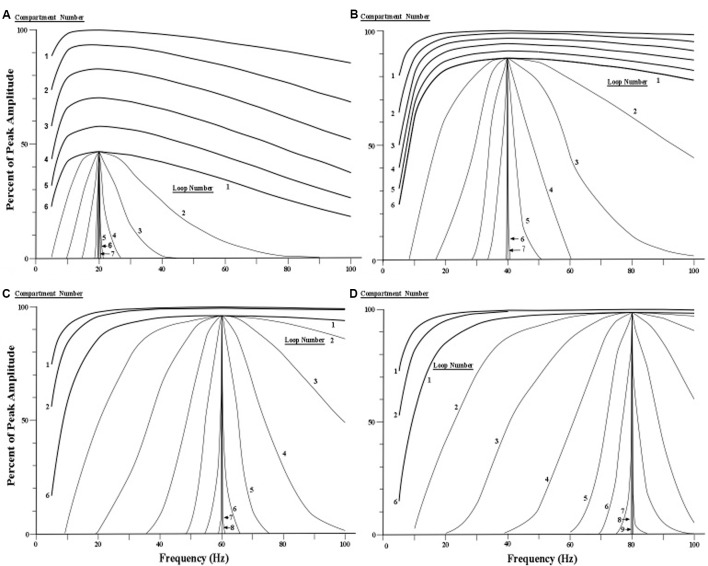
**Simulated narrowing of resonance curves. (A–D)** Resonance profile curves with peaks at 20, 40, 60, and 80 Hz are narrowed by cycling current repeatedly through six compartments of the apical dendrite via a circuit loop through the thalamus. The profile curves for the first six compartments are shown at the top of each of the four sets of curves (for clarity, the curves for the third, fourth, and fifth compartments are omitted for the 60 and 80 Hz conditions). The remaining curves in each of the four conditions show the effects of subsequent loops through the six compartments via the thalamus. Each curve that is labeled a loop is actually the output of the sixth compartment of each successive loop. [Adapted from Kasevich and LaBerge, 2011].

The four sets of profile curves produced by simulating the profile changes are shown in **Figure 7**. These curves are numbered first by each of the six compartments that the current surge first traverses, and then by the successive movements through all six compartments as the looping activity continues. Observation of the family of resonance profiles reveals that the amount of narrowing produced by moving pulses over the initial six compartments is very small, and hence there is scant indication of a peak in the curves. However, as the pulses move repeatedly through all six compartments a narrowing of the resonance profile is clearly observed, along with a distinct and consistent frequency peak located near the center of each profile. Also, by the seventh loop the width of the profile becomes less than 1 Hz for all four sets of profiles. The main difference in the four profile sets appears to be the number of loops needed to achieve a given width or spread of the profile. The profiles showing higher frequencies require more loops through the apical dendrite to narrow to a specific width.

We emphasize that the sets of profile curves shown in **Figure 7** are idealizations of the operations of the present model because synaptic noise is ignored in the simulations. The corticothalamic loop is assumed here to contain two synapses, one located where the pyramidal neuron’s axon contacts a thalamic neuron, and the other located where the thalamic axon contacts the pyramidal neuron at the top of the apical dendrite (see **Figure 2**).

If we assume that neural noise at each synapse, on average, adds a constant amount of impedance (opposition) for each frequency across the 1–100 Hz range, then each synapse will dampen or flatten the shape of the oscillation frequency profile. While every cycle of the corticothalamic circuit adds noise to flatten the shape of the profile curve, the next cascade through the apical dendrite compartments narrows the shape of the profile. If the total noise in the corticothalamic loop is sufficiently large relative to the number of apical dendrite compartments, then the resonance profile cannot be narrowed to the maximum asymptotes shown in **Figure 7**.

Therefore, increasing the number of compartments in the apical dendrite provides a means for effectively offsetting the synaptic noise in the corticothalamic circuit. Hence, longer apical dendrites (e.g., in primates) should produce more narrow frequency profiles, while very short apical dendrites (e.g., in mice) should show a limit on the narrowness of the resonance profile. For a comparison of apical dendrite lengths of layer 5 and layer 2/3 pyramidal neurons across five mammalian species see Figure 1 in LaBerge (2005).

In view of these considerations, it would seem that neural processing would be more precise when the number of compartments in an apical dendrite is large, so that the operations of profile sharpening can quickly produce a very small and stable profile spread around the peak frequency.

To summarize the main findings of the present simulation experiment, the relationship between outward potassium current and the peak frequency of apical dendrite oscillation is shown in **Figure 6** as a curve in which the peak frequency increases toward an asymptote as outward current increases. In **Figure 7**, the four sets of resonance profile curves show the establishment of four peak oscillation frequencies in the apical dendrite during early cycles of current in the corticothalamic loop. Moreover, they show that each profile around these peak resonance frequencies is sharpened to a width of less than 1 Hz by repeated cycling of electric surges through the apical dendrite.

## Two Tuning Neurons

### The Pyramidal Neuron of Layer 6

**FIGURE 8:**
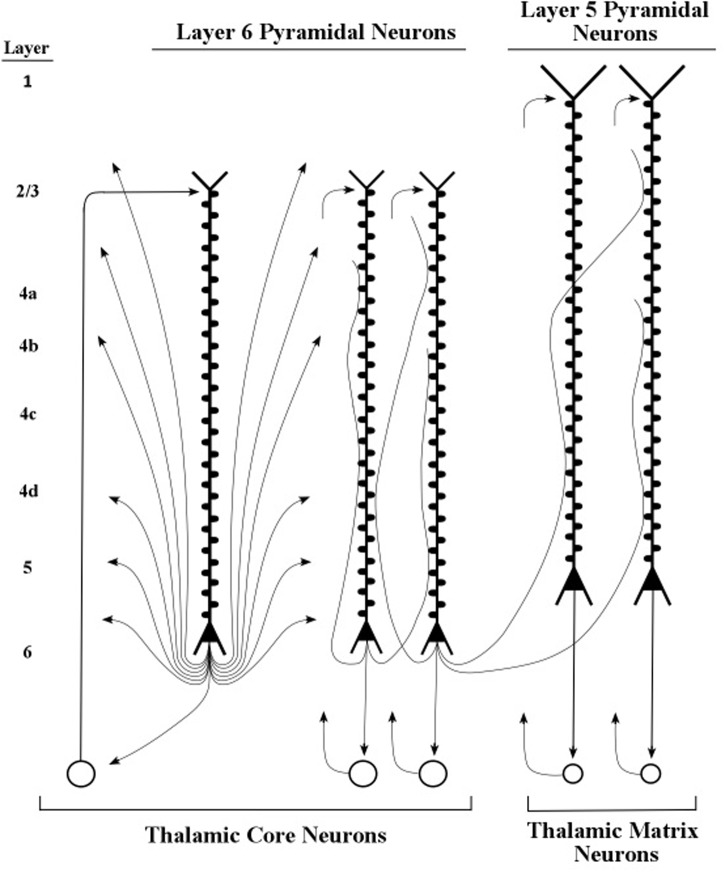
**Layers 5 and 6 pyramidal neurons and their axons**. Axons of layer 6 pyramidal neurons contact spines of layer 6 and layer 5 apical dendrites. Adapted from Nieuwenhuys et al. (2008, p. 564).

The present simulation findings suggest that pyramidal neurons that set the oscillation frequencies of other neurons have somas that are located in layers 5 and 6 of the cerebral cortex. However, we regard the long pyramids in layer 6 as the main tuning neurons because their axons contact the spines of layer 5 apical dendrites, while the axons of layer 5 pyramids apparently do not contact the spines of layer 6 apical dendrites (Thomson, 2010). The diagram in **Figure 8** shows axons of layer 6 pyramids contacting the spines of apical dendrites of layer 5 and layer 6 pyramids. Moreover, layer 5 pyramids with long apical dendrites can deliver bursts of axon spikes while layer 6 pyramids rarely produce bursts (Mercer et al., 2005; Llano and Sherman, 2009). Taken together, these considerations support the present hypothesis that the layer 6 pyramidal neurons serve as the basic tuning neurons.

**FIGURE 9:**
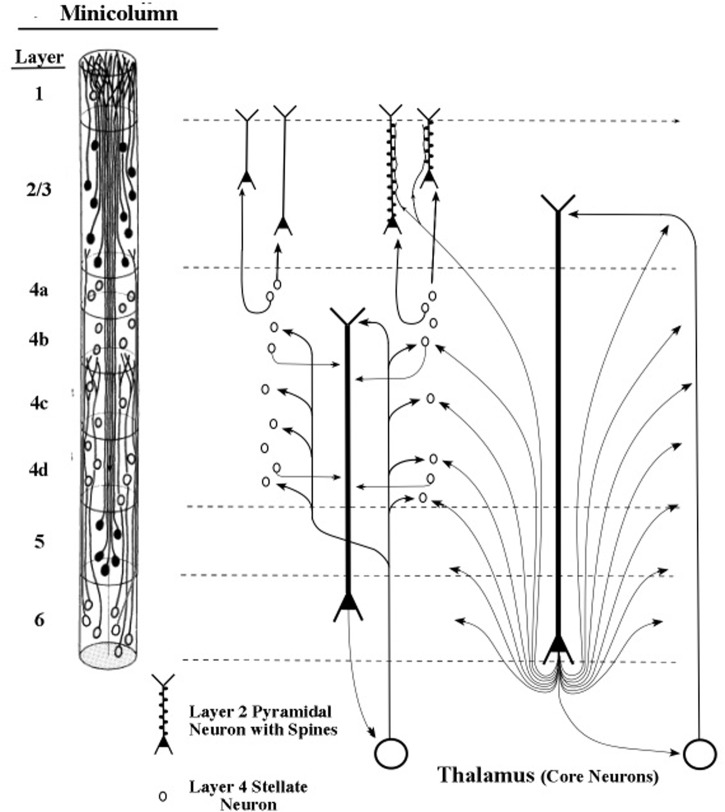
**Targets of cortical projections of thalamic core axons**. Direct thalamic projections target layer 4 stellate neurons with signals from sensory receptors. Indirect thalamic projections to the same stellate neurons are routed through the apical dendrites of layer 6 pyramids before they contact the stellate neurons (Sherman and Guillery, 2011). Minicolumn from primary sensory area of vision (Peters and Sethares, 1991).

**FIGURE 10:**
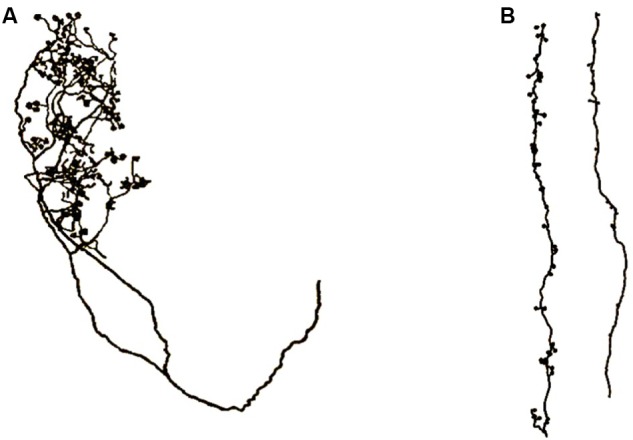
**Axons of core thalamocortical neurons. (A)** Axon projecting to layer 4 stellate neurons directly from the thalamus. **(B)** Two layer 6 pyramidal axons projecting to apical dendrite spines of layer 6 pyramidal neurons. Drawings made from light microscopic tracings by Sherman and Guillery (2011).

The pyramidal neurons of layer 6 are widely diversified in area V1 into at least eight different types, which are defined by their patterns of dendritic inputs and axon outputs (Briggs, 2010). Sherman and Guillery (1998) described particular layer 6 pyramidal neurons whose axons appear to modulate, as opposed to driving, the processing of neurons they contact (**Figures 9**, **10**). Examples of driver axons are the output fibers arising from thalamic neurons which directly contact stellate neurons of layer 4, and whose axons presumably produce spikes. Examples of modulatory axons arise from layer 6 pyramidal neurons and contact apical dendrites of layers 2/3, 5, and 6 pyramidal neurons as well as the stellate neurons of layer 4 (**Figure 9**).

Thalamic neurons that project to the cortex are of two main types, called “core” neurons and “matrix” neurons, which are distinguished by their immunoreactivity for the calbindin calcium-binding protein and the parvalbumin calcium-binding protein, respectively (Jones, 1998, 2007). The core thalamic neurons project to middle layers of the cortex, as illustrated diagrammatically in **Figure 9**, and the matrix thalamic neurons project to superficial layers 2 and 3 of the cortex (**Figures 13**, **14**), not only within a minicolumn or column but more widely across the cortex (Jones, 2002).

The findings of Sherman and Guillery (1998) support the view that the direct connection between the thalamus and a stellate neuron carries a series of spikes that codes the information arising from sensory receptors (e.g., in the retina), and in higher-order thalamic neurons codes the information arising from other cortical areas (Mitchell et al., 2014). In the context of the present theory, the connection between the layer 6 tuning-neuron and a stellate neuron provides the precisely timed intervals which adjust the series of spikes into a stable temporal code, although the details of this connection may not be as simple as the diagram in **Figure 9** suggests (see Mitchell et al., 2014). The temporally coded series of spikes is then sent to layer 2/3 where it contacts neurons in the cortical pathways of the horizontal network circuit. In this way, the information in the sensory receptor is transmitted to cortical networks in the form of a precise temporal code.

The presence of stellate neurons in layer 4 of a minicolumn serves as a marker of incoming fibers that send information-bearing signals from the thalamus, in the case of primary sensory areas, or in the case of higher order areas from layer 2/3 neurons of lower cortical areas (Anderson and Martin, 2009). These signals are then relayed to networks that pass horizontally across the receiving minicolumn. Some areas of the cortex contain many stellate neurons in layer 4 of their minicolumns and are called granular cortical areas, e.g., the primary sensory areas (Nolte, 1988). Some cortical areas have little or no stellate neurons in layer 4 and are called agranular areas, e.g., the motor area, some parts of area 6 (Brodmann, 1909/1994), and the anterior insular area (Morel et al., 2013). Other cortical areas that show intermediate quantities of stellate neurons in layer 4 are called dysgranular areas, e.g., the area 8 frontal eye field (Amiez and Petrides, 2009) and parts of the orbitofrontal area (Wallis, 2012). It would seem that cortical areas which are agranular support mainly loop activity (for the more complex motor area, see Shipp, 2005), and cortical areas which are granular support both network and loop activity. In particular, agranular areas which lack the horizontal network which passes through layer 2 (the Band of Bechterew, to be described in the next section), may function entirely in the loop mode (e.g., the anterior insula, Morel et al., 2013). These anatomical findings suggest that the presence of stellate neurons in layer 4 could serve as a marker of network processing in that area, and the absence of stellate neurons in layer 4 could serve as a marker for exclusively loop processing in that area.

**Figure 10** shows an axon that projects directly from the thalamus to layer 4 stellate neurons and it shows another axon that projects from a layer 6 pyramidal neuron to apical dendrites of layer 6 pyramidal neurons. In **Figure 9**, one can observe the different patterns taken by these axons as they make contact with their stellate or apical dendrite targets.

The axons in **Figure 10B** are vertically oriented, long and comparatively thin, which apparently enables them to fit between and follow the outside surfaces of the long, vertically oriented, apical dendrites as the dendrites extend toward layer 1. In contrast, the axons in **Figure 7A** are apparently configured to contact the scattered grouping of stellate neurons in layer 4.

Because the membranes of neurons that form a specific circuit in the networks of information processing oscillate at the same frequency, the neurons can receive coded pulse trains from each other. Thus, the frequency of oscillation acts like a frequency “channel” in two-way radio communications. The preference of these neurons for one particular frequency implies that spike communications coded at a different frequency will be blocked at the membrane.

Therefore, given these considerations, it would seem plausible that the frequency of coded pulses can serve as the basis for selecting particular cortical circuits, and for selecting the corresponding cognitive function(s) that they provide. In short, the layer 6 apical dendrites of a column cluster of minicolumns may serve to group neurons into functional circuits.

Although separate sets of layer 6 pyramids that oscillate at different frequencies select different circuits within the horizontal cortical network, many of these layer 6 pyramids and their selected circuits are presumably active at the same time. For example, retinal activity that registers the details of an entire visual scene eventually reaches visual cortex by parallel sensory (core) thalamic pathways, and each pathway is given its temporal code frequency by axon fibers from thalamic neurons both directly (via stellate neurons) and indirectly by way of apical dendrite oscillations of layer 6 pyramids (**Figure 9**).

For areas above the primary visual area (in which the thickness of layer 4 is reduced) the inputs to the core thalamic neurons include layer 6 axon inputs from lower areas (e.g., V2, A2, and S2) and axon inputs from frontal areas. This confluence of inputs to core as well as to matrix thalamic neurons is illustrated in**Figures 12**, **13** and treated in more detail below.

However, it is commonly assumed that cognitive processing of a visual scene usually requires that a part of the scene is selected for special processing by an appropriate circuit or a part of a circuit. This more restrictive selective activity is commonly called *attention*. The particular form of attention being treated here is called *sustained attention*, which can be contrasted with brief-acting forms of attention, such as typically found in visual search and orienting (LaBerge, 2002).

Here, we are treating the sustained activity as a basic function of corticothalamic loop circuits. However, when sustained activity functions as sustained attention, additional neural mechanisms appear to be involved. Here, we describe one of those neural mechanisms.

### The Bursting Pyramidal Neuron of Layer 5

As described in the previous section, an apical dendrite in a layer 6 pyramid loop communicates its particular oscillation frequency to a group of cortical neurons, which are then able to interact with each other by signals traveling on that particular “carrier frequency.” More specifically, when the membranes of dendrites and somas of two or more neurons oscillate at a common frequency (but at subthreshold voltages), only incoming axon spikes at that frequency will be accepted by the neurons.

We assume that the typical set of circuits selected by a layer 6 apical dendrite provides neural substrates for an array of operations related to a specific task, e.g., to a search task, a response planning task, or a perceptual preparation task. At any given moment during the execution of one of these tasks only a particular stage of the task may dominate processing, in which the corresponding circuit within the horizontal network shows increased amplitude.

One way to enable one neuron to dominate another neuron has already been described here for layer 6 axons. When two layer 6 axons compete for dominance at a dendritic or somatic membrane, the one whose spike frequency matches that of the membrane’s ongoing oscillation succeeds in delivering its series of spikes; if the other axon responds at some other spike frequency its input will be blocked. Conventional metaphors for this blocking type of selective mechanism are a *filter or gate*.

Another way to enable a particular axon to dominate other axons that synapse on a common dendritic segment is by shifting to the mode of bursting. When the signal amplitude of the target axon is sufficiently greater than that of other axons in the synaptic vicinity the signal inputs from other axons will be blocked. In electrical physics an appropriate metaphor is *gain (or voltage) control*, corresponding to the term e*nhancement* in psychology. Another cognitive metaphor for this amplifying type of selection is a *spotlight.*

**FIGURE 11:**
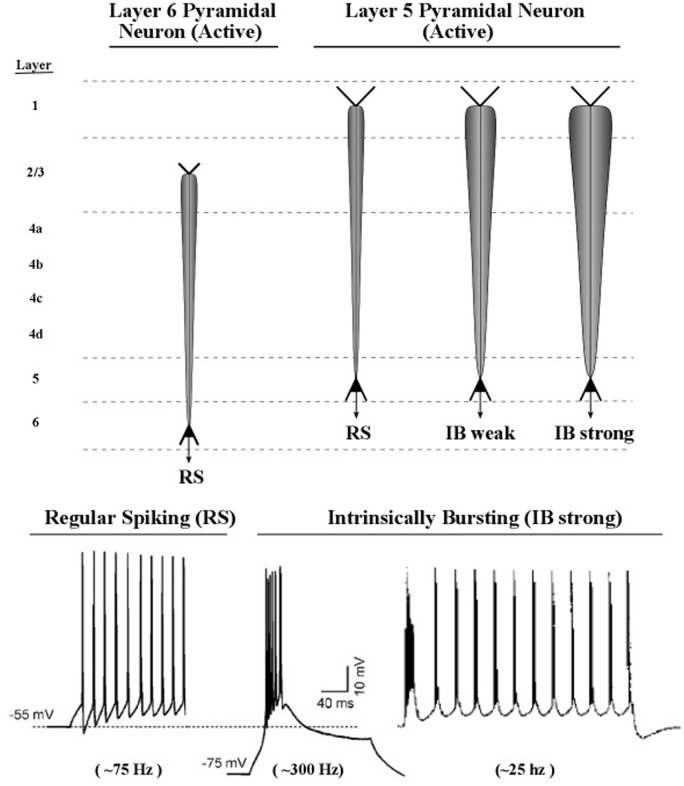
**Proposed graphic descriptions of regular spiking and intrinsic bursting neurons**. Layer 6 pyramidal neurons produce regular spiking, which is represented by a narrow carrot-shaped icon (intended to resemble the electric field of the active apical dendrite). The tapering carrot shape reflects the diminishing electrical effect of the persistent leak current, by which positive ions of potassium flow out of the dendrite. Layer 5 pyramidal neurons produce regular spiking, but also intrinsic bursting, which is a series of brief groups of very rapid spikes. As the number of spikes in a burst increase, the voltage of the burst pulse increases. To represent these differences in burst intensity the carrot-shaped icon varies in width.

Axon inputs to a neuronal membrane can vary in amplitude if more than one spike can be delivered in a very short interval of time, e.g., bursts of spikes at 200 Hz, at intervals of 50 ms. In practice, detecting a burst in a recording of axon spike trains is a complex process, owing to the great variety of burst patterns involving both inter-burst and intra-burst time intervals (Cotterill et al., 2016). The lower part of **Figure 11** shows examples of axonal spikes in neurons of both major types of layer 5 pyramidal neurons: regular-spiking neurons (RS) and intrinsic bursting neurons (IB).

Many diagrams of cortical circuits include pyramidal neurons, and it would seem convenient to use a graphic symbol or icon to indicate when the apical dendrite of a pyramidal neuron is oscillating, along with an indication of the amplitude of its oscillation. Use of this kind of graphic symbol would parallel the existing convention of representing the spike activity of an axon as a line containing a series of hatch marks.

Shown in the upper part of **Figure 11** are graphic representations of the oscillating apical dendrites of regular-spiking and bursting neurons. The typical electric oscillation activity of apical dendrites is represented as a carrot-shaped smearing of current surges along the apical dendrite. Thicker carrot-shapes correspond to higher amplitudes of oscillation in the apical dendrite.

**FIGURE 12:**
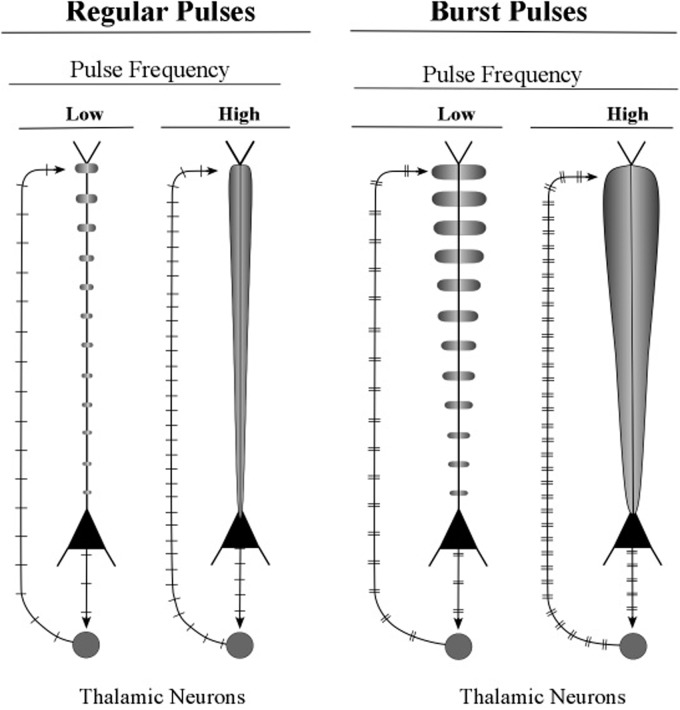
**Illustrative graphics of regular-spiking and bursting neuronal activity**. The figure shows the carrot-shaped graphics of apical dendrite activity, which depicts the shape of the electric field surrounding the apical dendrite. To the left of each carrot-shape is a vertical line with hatch marks which displays traditional recordings of axon spikes in regular spiking modes and bursting modes, weak and strong.

The graphic shapes which depict the apical dendrite oscillations of **Figure 12** (upper part) are outlines of the series of current surges shown in **Figure 11**. Each pulse represents a surge of current that is injected at the top of the apical dendrite by axons arising from the thalamus. For the layer 6 pyramidal neurons, the source of the axon inputs is a core thalamic neuron; for the layer 5 pyramidal neurons the source is a matrix thalamic neuron (Jones, 2002, 2007). Axons of thalamic matrix neurons tend to contact pyramidal neurons within the minicolumn that originally sent axons to the thalamic matrix neurons, but they also contact pyramidal neurons in minicolumns that are located at adjacent cortical areas. Axons of core thalamic neurons tend to contact neurons located within the cortical column of origin, which is a large bundle of ∼100 minicolumns in primates (Mountcastle, 1998).

**FIGURE 13:**
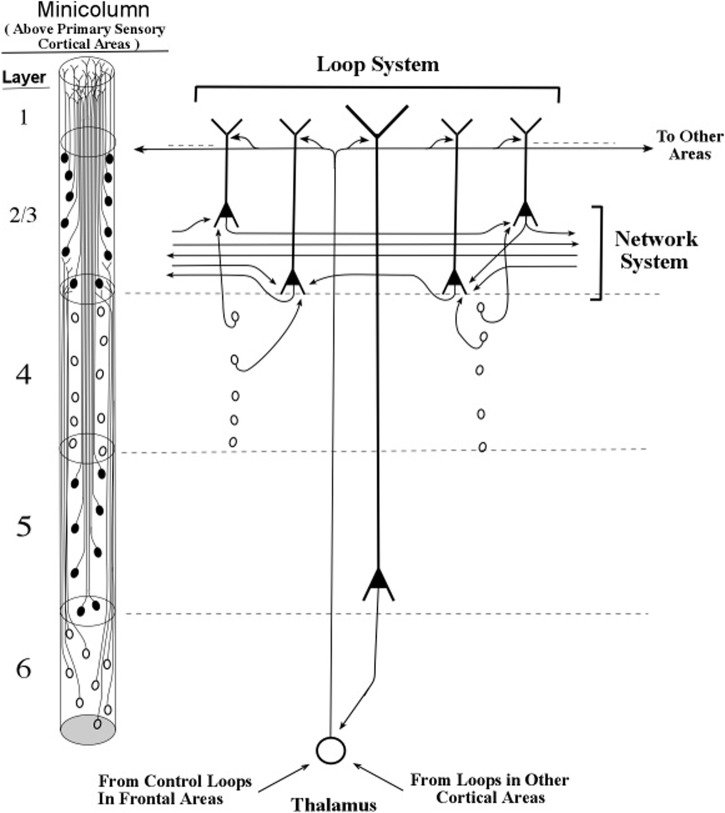
**Intersections of inactive layer 5 loops and an inactive network system within a minicolumn**. The thalamocortical input to the networks system is itself influenced by loops located near and far, as shown in the bottom-up and top-down arrows that contact the thalamic neuron shown in this figure. Informational pulse inputs to the networks system are indicated by axons from stellate neurons. On the left side of this figure, the diagram for minicolumns in higher order cortical areas indicates the vertical extent of layer 4, which is considerably shorter than in previous figures based on the primary sensory cortex of vision.

Small bundles of layer 5 pyramids form the central core of the minicolumn, and **Figure 13** shows the major connections of one bursting pyramidal neuron. The major input to the apical dendrite of this layer 5 pyramid is the thalamic axon, which enters at the distal segment at the top. Here, the axon branches and the branches leave the layer 5 loop and make contact with the top of apical dendrites of layer 2/3 pyramids. As a consequence, the apical dendrites of layer 2/3 pyramids will engage in oscillating activity that matches the oscillation frequency of the layer 5 pyramids. The oscillations of the layer 2/3 pyramidal neurons will also be fine-tuned, because they received the outputs of the long layer 5 neurons, whose longer apical dendrites have produced the refinement process of fine tuning. When two layer 2/3 pyramidal neurons, separated by small or large cortical distances, oscillate in synchrony, they can effectively communicate with other. In this way, the layer 5 pyramidal axons (shown in **Figure 13**) can increase the spatial spread of a synchronous circuit of layer 2/3 neurons within the horizontal network. Layer 2/3 neurons which show high-frequency bursting activity have been named *chattering cells* (Gray and McCormick, 1996).

With the apical dendrites of layer 2/3 pyramids oscillating at the same frequency as the layer 5 pyramids, and with the layer 6 pyramids setting the oscillation frequency of the layer 5 pyramids, all of the pyramidal apical dendrites of the minicolumn apparently oscillate in synchrony. The layer 4 stellate neurons emit spikes within the frequency band of the minicolumn oscillation frequency because their neural membranes receive subthreshold pulses originating from layer 6 axons. Hence, it would appear that under typical conditions membranes of all of the excitatory neurons of a minicolumn oscillate in synchrony at a particular frequency. Moreover, since the column is defined by being activated by the same group of layer 6 pyramidal axons, the entire column of excitatory neurons oscillates at a specific frequency.

The “horizontal” fibers in **Figure 13** (and in the figures that follow) represent the cross-cortical connections in the part of lower layer 2/upper layer 3 called the Band of Bechterew (Gray, 1918). Another cross-cortical line of fibers observed at the minicolumn level is the Band of Baillarger (Gray, 1918), which has two parts, one lying in layer 4 and one lying in layer 5. Other cross-cortical fibers are the many long-distance fibers that appear to “jump” between locations separated by large distances, which gives a two-dimensional map of the brain the appearance of a map of airline routes across the United States or Europe. For purposes of presenting a clear description of the network connections of long apical dendrites, we restrict our present treatment of the horizontal network fibers to the Band of Bechterew lying in lower level 2/upper layer 3.

**FIGURE 14:**
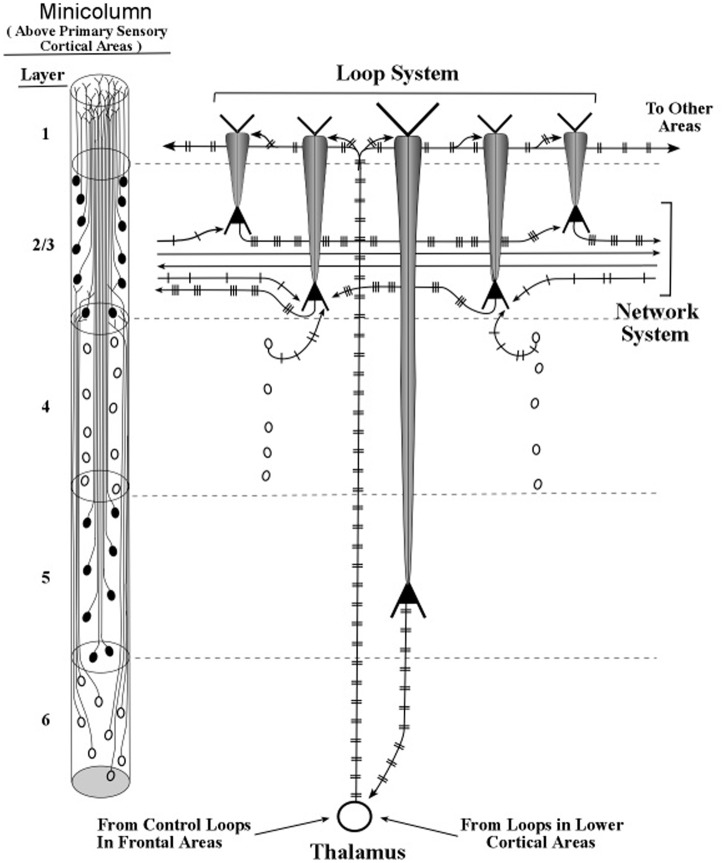
**Intersections of active layer 5 loops and networks systems within a minicolumn**. The loop and network diagrams in **Figure 13** are shown here with patterns of burst firing. Loop inputs and outputs are presumed to be rate-coded, while network inputs and outputs are presumed to be mostly temporally coded.

The excitatory neurons in the networks within the Bechterew Band consist of pyramidal axons whose somas lie in layer 2 and 3. Unlike the taller pyramidal neurons of layers 5 and 6, the layers 2 and 3 pyramidal neurons are not part of a loop circuit. Instead they act as extensions of the layers 5 and 6 loops that enable the oscillations within a minicolumn to influence the spike signaling in the horizontal networks (**Figures 9**, **13**). Clearly, when an axon from a layer 5 loop shifts a layer 2/3 pyramid from regular-spiking to bursting the input–output of that layer 2/3 pyramid will be enhanced (see **Figure 14**). Thus, oscillations in a minicolumn can affect the input–output operations within layer 2 and layer 3 pyramids.

In a previous publication (LaBerge, 2005), this interaction between apical dendrite activity and soma input–output processing was described as a modulatory action in which amplitude of the apical dendrite changes the threshold of firing at the soma. Higher amplitude oscillations at the soma allow weaker input signals to evoke spikes in the axon, while low amplitude oscillations require strong input signals to evoke spikes. In effect, higher amplitude oscillations in layer 2/3 pyramids increases the signal-to-noise ratio of an input at basal dendrites. As a result, the latency of the input–output response is lowered and a given signal input can be effective when neural noise is increased.

Sufficiently high amplitudes of oscillations in layer 2/3 pyramids often represent a high level of preparatory attention, as for example arises from a high expectation that one particular event will be followed by another event (e.g., LaBerge et al., 2000). A high level of preparatory attention sometimes produces premature responses (“jump the gun” responses), which are based on axon outputs that occur without the associated input. A familiar example is the driver who starts to move a car at an intersection just before the green light appears.

Attention-based upward adjustments of amplitudes of oscillations in layer 2/3 pyramidal neurons apparently are not needed for an input in the basal dendrites to evoke a response in the axon when a task is learned to a high level. We customarily label the performance of these tasks as “automatic,” requiring little or no attention. Examples are writing one’s name, tying shoelaces, and adding single-digit numbers. Adding numbers of 2 or more digits usually requires attentional assistance, and, according to the present model, this involves amplifying the oscillations in the layer 2/3 pyramidal neurons so that their somas are more sensitive to weaker inputs at their basal dendrites. The amplification is assumed here to be produced by axons arising from layer 5 loops and terminating on apical dendrites of layer 2/3 pyramidal neurons (see **Figure 14**).

Thus, the transfer of neural activity from layer 5 amplifying loops to the horizontal network circuits results in adjustments of response thresholds at the somas of layer 2/3 pyramids, while the transfer of neural activity from layer 6 tuning loops to apical dendrites of layers 2/3, 5, and 6 pyramids involves only tuning adjustments of spike frequency at synapses of spines located along the apical dendrites of these pyramids.

The transfer of neural activity from loops of oscillating layer 5 pyramids to action potentials in circuit networks (via oscillations in layers 2/3 pyramids), may be made more explicit in **Figure 14** with the addition of spike trains to the diagram in **Figure 13**.

The patterns of spikes within a burst are apparently highly stereotyped and very robust to noise (Kepecs and Lisman, 2003). Compared to the timing of isolated spikes in regular-spiking neurons, the timing between bursts is much more reliable (Silva et al., 1991). This difference may be expected because, according to the present theory, the fine-tuning of layer 5 apical dendrites during looping activity is based on axons of layer 6 neurons, which have already received fine-tuning by their own looping activity. Therefore, the layer 5 pyramidal neuron is expected to be particularly successful in maintaining a relatively constant interval between bursts.

In addition to increasing the reliability of signals, the spike pattern of a multi-spike burst carries a large amount of information. Bursts in thalamic axons occur during waking states (Marlinski and Beloozerova, 2014) and they could transmit information about external stimuli (Sherman, 1996; Lesica and Stanley, 2004) which apparently is based on the *n*-spike burst code (Elijah et al., 2015). This *n*-spike coding may be a general property of thalamic neurons and it is possible that interspike frequencies could resonate with subthreshold membrane oscillations of a postsynaptic neuron (Izhikevich et al., 2003), which would enable the kind of selective communication between neurons described in the present paper. However, the present model treats issues of the tuning and tuning-refinement by describing the underlying neural mechanisms in apical dendrites. For example, these apical dendrite mechanisms respond to layer 6 axon inputs to set the resonant frequency value of their oscillations, and they also refine that value to less than 1 Hz by repeatedly passing EPSPs along the apical dendrite via the thalamocortical loop.

We have described how bursting in axon inputs to the distal segment of apical dendrites of layer 2/3 pyramidal neurons could increase the tonic level of voltage at the soma so that only a small increase in current from a basal dendrite is required to produce a discharge into the axon This amplification effect at the soma resulting from facilitating basal dendritic input has been recently described as the Apical Amplification (AA) hypothesis by Phillips (2017). The dendritic tuft apparently represents a separate segment of the apical dendrite that functions as a subthreshold integrator of tuft inputs which has an augmenting effect on synaptic inputs located on basal dendrites.

The idea that synaptic inputs at the apical dendrite tuft can produce a modulating effect on synaptic inputs throughout the layer 5 pyramidal neuron was put forth by Schwindt and Crill (1995). They examined the effect of specific channel blocking agents on the current arriving at the soma during iontophoresis of glutamate at a distal site on the apical dendrite and found evidence for amplification of input current at dendritic synapses.

Schiller et al. (1997) reported that local calcium action potentials, which were subthreshold, can amplify the tufts of the distal apical dendrite. They concluded that layer 5 neocortical pyramidal neurons have a functionally separate dendritic zone enriched with active conductances for integrating and amplifying distal synaptic inputs. Because this tufted segment of the layer 5 pyramidal neuron contains active conductances for integrating and amplifying synaptic inputs it therefore serves a function separate from the rest of the apical dendrite. That function appears to be modulating the effectiveness of other synapses in the pyramidal on the subthreshold activity in the dendritic membranes of the dendrite. In the present theory, it is assumed that this layer 1 amplification function operates not only in the long layer 5 pyramidal neurons but also in the neighboring shorter layer 2/3 apical dendrites whose tufts interweave closely with those of the layer 5 tufts. Zhu (2000) also found that active calcium synapses contributed to the amplification effect in layer 5 pyramidal neurons.

The close interweaving of the tufts of layer 5 apical dendrites with tufts of many layer 2/3 apical dendrites surrounding each layer 5 tuft, plus the relatively wide horizontal spread of each layer 5 apical dendrite tuft implies that together the tufts extend over many minicolumns and even columns. This clustering of tufts may resemble a canopy, under which many more layer 6 pyramidal neurons have room to stand, given that their apical dendrites are shorter than those of their neighboring layer 5 pyramidal neurons.

Up to this point in the present study, the only excitatory inputs to the layer 1 segment of the apical dendrite are axons arising directly from the thalamus within the corticothalamic loop circuit. The foregoing descriptions of a possible function of the apical dendrite adds another source or sources for excitatory inputs to layer 1 of the apical dendrite.

The tufted top layer of layer 5 pyramidal neuron receives axon fibers from many cortical sources, most of them (90%) as connections from distant locations and less of them (10%) as connections from locations nearby (Hubel, 1982). An early study by Cauller et al. (1998) confirmed that layer 1 of the somatosensory area (S1) received fibers from a variety of areas located at a distance from area S1, including the primary motor area, the lateral parietal areas, and the agranular insular cortex. In a survey of relevant studies of this topic, Larkum (2013) claimed that the pyramidal neuron acts to couple feed-forward and feedback information at the cellular level of the cortex. This additional function of the apical dendrite is compatible with the present model if the set of inputs to tufts of a particular pyramidal apical dendrite share the same carrier frequency provided by axons of layer 6 pyramidal neurons.

To summarize the main points of this section, the thalamic inputs to the top of the long apical dendrites of layer 5 pyramids can consist of trains of bursts, unlike the inputs to the top of long layer 6 apical dendrites, which almost always consists of the regular firing of single spikes. Each burst is regarded as a surge of current, but its voltage is higher than the voltage of the current produced by a single spike input to the apical dendrite. As these high-voltage surges repeatedly pass down the apical dendrite via the corticothalamic loop, the apical dendrite oscillates at a high voltage. While being fine-tuned by passing repeatedly down the apical dendrite, these oscillating pulses are transmitted to the adjacent pyramids of layer 2/3. There, they produce an elevated tonic level of voltage at the somas, which effectively lowers the amount of voltage at basal dendrite synapses needed to evoke a spike response at the soma. This lowering of the response threshold allows inputs at basal dendrites to evoke spike responses in the layer 2/3 neurons when they previously could not. In this way, burst-firing of layer 5 pyramidal neurons can selectively enhance the processing of particular circuits within the horizontal network.

The circuit effects of burst firing in layer 5 pyramids can be illustrated by the cognitive example of the “hidden image” of a Dolmatian dog in a degraded scene of light and shadows. Typically, an observer reports an array of black forms within a white background, but soon the figure of the dog suddenly appears in the foreground. The present theory claims that the first perception is produced by outputs from layer 6 loops, which produce trains of single spikes to neighboring apical dendrites of layer 2/3 pyramids. The enhanced spikes of the layer 2/3 neurons are then transmitted to their axons within the horizontal network of cortical processing. The second perception (the shape of the dog) is produced by inducing a group of layer 5 neurons, representing a part of the whole scene, to fire bursts of spikes. Without accounting for the detailed way that this selection is made, one can infer that the selection of the dog’s shape and the location of the dog’s shape occurs at virtually the same time, and one can infer that the supporting circuits for these two events dominate other circuits that might be concurrently active.

## Control of Frequency Tuning

The selection of particular circuits to dominate momentary the ongoing neural cognitive activity is necessary for the selection of an effective adaptive response to a situation. One reason for circuit selection is that the nervous system can produce many alternative responses and many of these responses have topologies that conflict with each other.

The present model describes two major types of visual circuits that are typically selected during the waking state. One is broad in scope and the other is narrow in scope. The first type of selected circuit addresses the broad visual scene when its neurons are activated by core thalamic neurons that evoke oscillations in apical dendrites of layer 6 pyramids; the second type addresses a part of the ongoing visual scene when its neurons are induced by matrix thalamic neurons to fire in bursts, which evokes higher-amplitude oscillations in apical dendrites of layer 5 pyramids.

The first selection (of the broad scene) involves selecting a particular frequency at which the large (column-wide) set of layer 6 pyramids oscillate. The second selection (a part of the scene) consists of a nesting set of layer 5 pyramids, that will oscillate at the same frequency, because they are regulated by the same large set of layer 6 pyramids. In this way, the processing of the “whole” aspect of a topic is shifted to processing of a “part” of that topic, whether that topic is a sensory perception, an action plan, or a conceptual issue.

An example of processing a part of a conceptual topic is given by the diagrams in **Figures 13**, **14**. At the bottom of the two diagrams is shown two inputs to the thalamus from two different sources. One input arises from axons located within columns of earlier cortical areas, the other arises from axons located within columns of the frontal cortex areas of executive control. It is the input from the frontal control areas that mediates higher-order selection of the frequency with which apical dendrites of layer 5/6 pyramids will oscillate within their corticothalamic loops shown in **Figures 13**, **14**. These frontal cortical areas also control the burst-firing modes (number of spikes in each burst) of layer 5 pyramids.

**FIGURE 15:**
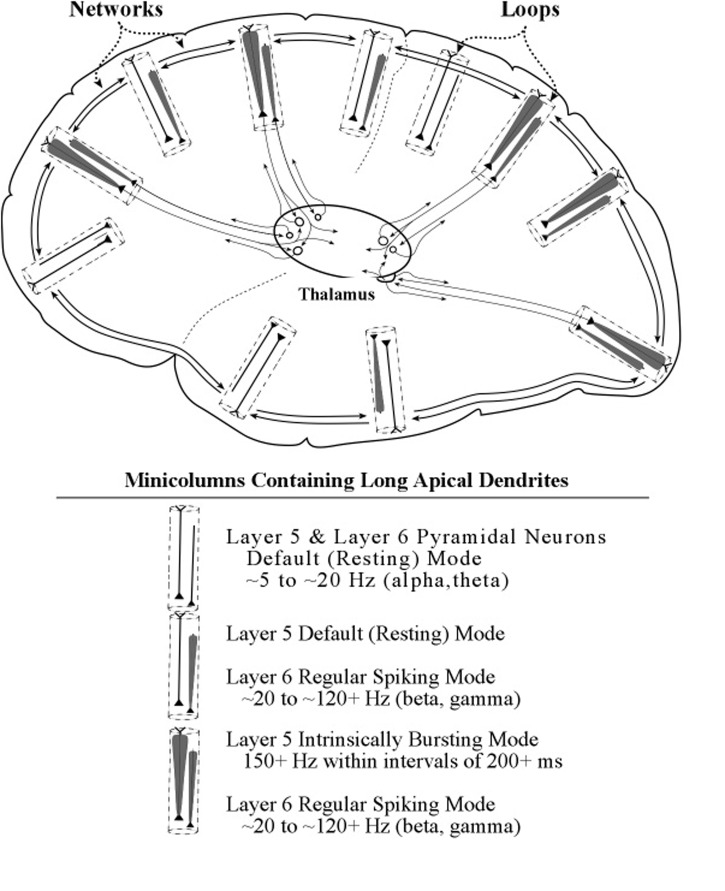
**A summarizing diagram of the loop and networks systems and some of their interactions**. The network system depicted is only one of the many linkages that extend across cortical minicolumns and columns. Within this figure is a diagram showing connections between the prefrontal area of voluntary control and the premotor area containing circuits for action plans. Within the thalamus are shown the axon connections of each layer 5 pyramidal neuron to 2 thalamic neurons. Axon connections of layer 6 pyramidal neurons show a similar pattern but are omitted here for the purpose of clarity.

Support for this pattern of linking two cortical sites, one serving as the “controller” and the other as the “controlled,” is provided in a study by Zikopoulos and Barbas (2007). The two cortical sites used in this study were the prefrontal cortex and the premotor cortex, which contain areas known for attentional control and planning of cognitive and motor actions, respectively. Combining the diagrams of layers 5 and 6 pyramid linkages to the thalamus (**Figures 9**, **13**) produces a diagram in which each of these two pathways connects the thalamus to the prefrontal cortex and each connects the thalamus to the premotor cortex (see **Figure 15**).

In the Zikopoulos and Barbas (2007) study, bidirectional chemical tracers were injected into prefrontal and premotor areas, and also into their common thalamic projection area, the ventral anterior nucleus. Separation of layer 5 and layer 6 projection neurons was possible owing to their neurochemical markers calbindin and parvalbumin (Jones, 2002), respectively. The results showed that in the ventral anterior thalamic nucleus the terminals of layer 5 and layer 6 axons emerging from the prefrontal and premotor areas intermingled or had overlapping sites. It may therefore be concluded that the prefrontal control areas are connected to premotor areas of action planning through the ventral anterior thalamic nucleus, as shown within the thalamus diagram of **Figure 15**.

In the context of the present model these results suggest that prefrontal areas can control which action plan is momentarily selected by projecting specific spike frequencies to premotor areas along layer 6 axons. Following the selection of an action plan, specific components of the plan can be selected to receive special processing by projecting bursts of spikes along layer 5 axons to the circuits in the part of the pre-selected premotor area that serve a component of the action plan. Womelsdorf and Everling (2015) describe specific ways that components of a plan can be selected (e.g., in the prefrontal cortex) with low frequency oscillations which are then projected onto high-frequency circuits in distant areas (e.g., in the parietal cortex), for appropriate processing.

Evidence for the influence of thalamic axons on transmission of sensory information to the cortex is usually based on anatomical connections between relevant regions (e.g., see Saalmann and Kastner, 2011). However, detailed descriptions of the *in vivo* neural effects of these connections have been given only recently. Two articles, Mease et al. (2015) and Castejon et al. (2016), report thalamic axons that amplify and prolong sensory inputs to areas S1 and S2 of rodents. The controlling thalamic axon that they studied originates from the posteromedial thalamic nucleus (POm) of the rodent, which is believed to correspond to the anterior pulvinar nucleus of the monkey. The primary sensory pathway arises from whisker barrel cortex. Mease et al. (2015) used optogenetic techniques to control the POm pathway and Castejon et al. (2016) used electrophysiolocial and pharmacological techniques. In particular, Mease et al. (2015) found that single pyramidal neurons in layer 5 integrate the outputs of the POm and the sensory pathways. The foregoing results appear to be consistent with the present theoretical hypothesis that the burst-firing of layer 5 pyramidal neurons enhances both oscillations and pulse signals (see **Figure 14**).

Layer 5 burst-firing and burst-synchronization may provide ways to coordinate attention across brain areas. A study by Womelsdorf et al. (2014) recorded cellular activity in anterior cingulate cortex and lateral prefrontal cortex (ACC/PFC) of macaque monkeys during a selective attention task. When the subjects began to attend to the task, neurons in the ACC/PFC increased their firing of brief 200 Hz spike bursts. Burst spikes showed synchrony over long cortical distances; indicating, in particular, that circuits in area 24 (in ACC) and 46 (in PFC), important to attentional control, were synchronized. These results support the hypothesis that burst-firing can produce the selection operation of attention by amplifying neuronal activity.

## Discussion and Summary

Neurons of the cerebral cortex appear to be temporally regulated in two important ways. The first way sets the frequency with which the membrane of the neuron oscillates. Circuits of these neurons can then communicate with each other on that “carrier” frequency, while incoming signals on other frequencies are blocked. The second way of tuning refines the frequency setting so that the momentary variability of frequency is near zero, thereby assuring that the duration of each oscillation cycle (clock cycle unit) is very close to constant. This fine-tuning produces a stable time unit that supports the use of temporal coding in spike trains sent between neurons. The temporal regulation of signaling in circuits of neuron resembles the effect of a clock that regulates the timing of operations within a computer.

The first tuning hypothesis concerns the initiation or setting of the oscillation frequency. In a recent publication (LaBerge and Kasevich, 2013) it was proposed that the regulation of the frequency of oscillation is produced by the long axon of a layer 6 pyramidal neuron through its synapses on the spines of the apical dendrite. The spike frequency of the layer 6 pyramidal axon at apical dendrite spines determines the average level of voltage on the inside of the membrane. This local voltage in turn regulates membrane gates that release potassium ions from the interior of the apical dendrite.

Support for this tuning (or resonance) hypothesis was provided by results of the simulation study of the model (Kasevich and LaBerge, 2011). The results showed that for oscillation frequencies of 20, 40, 60, and 80 Hz, the rate of outward potassium flow at the membrane increased monotonically with frequency.

This finding supports the first tuning hypothesis, which states that the setting of peak resonance frequency level in the apical dendrite can be produced by the spike rate of the layer 6 pyramidal axon which contacts the spines along the apical dendrite.

Peak resonance, indicated by a maximum in the impedance amplitude curve, is derived in the present model without using the inductive component featured in traditional electronic circuitry. However, in an earlier model of apical dendrite activity (Narayanan and Johnston, 2008) an induction-like component is provided by the operations of the membrane h channel. They supported their model by measuring impedances from the apical dendrite of a hippocampal neuron. However, owing to the timings of the h channel operations, their predicted peak resonances are restricted to the range of frequencies less than 20 Hz (which is appropriate for the range of typical measurements of hippocampal frequencies). For the present apical dendrite model, the range of predicable peak resonance ranges from 0 to 100 Hz and higher, which is appropriate for measurements of neocortical frequencies.

The second tuning hypothesis, which concerns the stability of the oscillation frequency (or the equivalent cycle duration), was also confirmed by the simulation findings. With each consecutive passage of the current surges around the corticothalamic loop, the distribution or profile of oscillation frequencies becomes more narrow. With a few additional loops the width of the profile became less than 1 Hz for each of the 20, 40, 60, 80 Hz oscillation conditions.

These simulation results are based on an idealized model in which there is no consideration of the effects of noise in the corticothalamic loop. The two main synapses in the loop presumably insert noise into the current surges, which perturbs the process of narrowing the resonance profile. Noise will have the effect of damping the oscillations, which flattens the profile. However, we reason that longer apical dendrites perform more narrowing operations on the moving surges within the dendrite without adding the noise from more synapses. Hence, longer apical dendrites should result in less noise and more profile narrowing with each cycle through the corticothalamic loop. But when synaptic noise is sufficiently high relative to the profile narrowing in each loop cycle, then no progress toward narrowing can take place, and consequently the clock time unit may remain unstable.

The present treatment of the functions of long apical dendrites of pyramidal neurons lead us to propose a separate neural system that complements the conventional information-processing view of cortical activity. We label the proposed system the *loops system*, in view of the geometrical form of its anatomical circuits. We label the traditional information-processing system the *networks system*, in consideration of its general mesh-like appearance with the many complex interconnecting links.

The diagram in **Figure 15** provides a graphic illustration of the loops and networks systems and their interactions. The electric oscillation activity on apical dendrites in the loops system is represented as a carrot-shaped smearing of current surges along the apical dendrite. The narrowing of the oscillation outline represents the effect of outward flow of ions as the current surges progress toward the soma. The electric spiking activity on axons in the networks system is represented in earlier figures in the conventional way, a series of hatch marks along a line that connects one neuron to another neuron.

The apical dendrites in the loops system bring with them an electric activity that differs sharply from the network system’s flow of electric current and voltage spikes traveling along fibers of varying length. The characteristic electrical activity of apical dendrites consists of oscillations of current surges at relatively low voltage levels that are subthreshold for the generation of spikes. These electric vibrations do not appear to be traveling anywhere, like spikes travel from one location to another within a network. Instead they stay in one place within a circuit and repeat their electric oscillations along their apical dendrite. The extended repetitions of electric vibrations in one place is made possible by the apical dendrite being a part of a loop circuit. In view of these considerations, a metaphor for the apical dendrite-in-a-loop would be a *container*, while the metaphor for the axon in a network would be a *conduit.*

### Interactions between Loops and Networks Systems

Spikes travel along fibers of network systems, while oscillations stay within their loops, but it is the interactions between loops and networks that underlies the theorizing of the present paper.

The first kind of interaction is produced by the layer 6 pyramidal axons, which tune the frequency of the stellate neurons to “carry” the spike information. As a result, the axons of the stellate neurons can relay spike-coded information from the thalamus to the layer 2/3 neurons in the horizontal network that passes through the upper part of the minicolumn (see **Figures 9**, **13**). The layer 6 pyramidal axons also tune the apical dendrites of layer 2/3 neurons to synchronize their oscillations with other fibers of the minicolumn (see **Figure 9**).

The second kind of interaction is produced by thalamic axons in the layer 5 loop, which increase the amplitude of pulse signaling in these networks by contacting the layer 2/3 pyramidal neurons at the top of their apical dendrites (see **Figures 13**, **14**). When the thalamic neurons of the layer 5 loop deliver bursts of spikes to the apical dendrite of layer 2/3 neurons the ongoing voltage at their somas is increased and incoming spikes at their basal dendrites produce bursts in their output axons. As a result, the pulse signals in the network circuits are amplified and serve as the dominant input to other neurons in the network.

The influence of the oscillations in the layers 5 and 6 pyramidal neurons on network circuits makes possible successful computation in neurons that receive spike inputs from other neurons within a circuit. The processing of a computation is presumed to be a momentary event, which requires that the arrival times of the component inputs be temporally coordinated in time. The present study supports the hypothesis that the oscillations of apical dendrites can provide the frequency tuning and frequency fine-tuning that are necessary for this neural computation to take place.

### Columns, Minicolumns, and Somato-dendritic Minicolumns

The present treatment of tuning by the apical dendrite is carried out within the framework of a cortical minicolumn. This version of the minicolumn is based on a small volume of visual cortex that contains neurons serving visual orientation (Peters and Sethares, 1991). The minicolumn is shown diagrammatically on the left side of many figures of the present paper to serve as a guide to the location of neuronal somas and apical dendrites that lie in arrays orthogonal to the 6 horizontal layers of the neocortex. Pyramidal neurons form the majority of cells in these arrays, and they are observed throughout the cortex in humans and all other species of mammals, along with their bundles of apical dendrites.

It was proposed by Mountcastle (1957) that a vertically organized column of neurons serves as the fundamental processing unit of the cerebral cortex. This concept has endured to the present time, even though no exact definition of a “column” (or minicolumn) was broadly accepted across the neuroscience community (Jones, 2000; Horton and Adams, 2005; Rakic, 2008; Rockland, 2010). In spite of its vagueness the concept of a vertical unit that repeated itself across the cortex apparently was kept alive by its descriptive usefulness to neuroscience investigators.

More recently an even smaller vertical unit was proposed by Innocenti and Vercelli (2010). In an earlier study (Vercelli et al., 2004), bundles of apical dendrites were found that are made up of neurons with specific targets. The authors describe bundles of these specific neurons as the “cortical output unit,” i.e., “an assembly of bundles of apical dendrites and their parent cell bodies including each of the outputs to distant cortical or subcortical structures, of a given cortical locus (area or part of an area)” (Innocenti and Vercelli, 2010).

Illustrative diagrams that may approximate a neuron in the “somato-dendritic minicolumn” are shown in **Figure 15** of the present paper. The column diagrams in this figure contain a pair of pyramidal neurons, each showing its triangular cell body, apical dendrite (with varying levels of activity), and an output axon (with a short length as an abbreviation of its actual length). One of the pyramidal neurons has its soma in layer 6, whose axon targets are the thalamus (core cells), stellate cells of layer 4, and the spines of apical dendrites (see **Figure 9**). The other pyramidal neuron has its soma in layer 5 with its axon targets being the thalamus (matrix cells) and the basal dendrites of layer 2/3 pyramidal neurons (see **Figure 13**). The connection of the layer 5 pyramidal neuron to the apical dendrites of layer 2/3 pyramidal neurons is indirect, through the thalamus in the thalamocortical loop, and axon targets are the thalamus (matrix cells) and the basal dendrites of layer 2/3.

## Author Contributions

DL drafted the manuscript in discussion with RK. RK edited the manuscript.

## Conflict of Interest Statement

The authors declare that the research was conducted in the absence of any commercial or financial relationships that could be construed as a potential conflict of interest.

## References

[B1] Amiez C. Petrides M. (2009). Anatomical organization of the eye fields in the human and non-human primate frontal cortex. *Prog. Neurobiol.* 89 220–230. 10.1016/j.pneurobio.2009.07.010 19665515

[B2] Anderson J. C. Martin K. A. C. (2009). The synaptic connections between cortical areas V1 and V2 in macaque monkey. *J. Neurosci.* 29 11283–11293. 10.1523/JNEUROSCI.5757-08.2009 19741135 PMC6665918

[B3] Briggs F. (2010). Organizing principles of cortical layer 6. *Front. Neural Circuits* 4:3 10.3389/neuro.04.003.2010 PMC2826182 20179784

[B4] Brodmann K. (1909/1994). *Brodmann’s Localisation in the Cerebral Cortex, L J. Garey (Translator)*. New York, NY: Springer.

[B5] Castejon C. Barros-Zulaica N. Nunez A. (2016). Control of somatosensory cortical processing by thalamic posterior medial nucleus: a new role of thalamus in cortical function. *PLoS ONE* 11:e0148169 10.1371/journal.pone.0148169 PMC4731153 26820514

[B6] Cauller L. J. Clancy B. Connors B. W. (1998). Backward cortical projections to primary somatosensory cortex in rats extend long horizontal axons in layer I. *J. Comp. Neurol.* 390 297–310. 10.1002/(SICI)1096-9861(19980112)390:2<297::AID-CNE11>3.0.CO;2-V 9453672

[B7] Cotterill E. Charlesworth P. Thomas C. W. Paulsen O. Eglen S. J. (2016). A comparison of computational methods for detecting bursts in neuronal spike trains and their application to human stem cell-derived neuronal networks. *J. Neurophysiol.* 11 306–321. 10.1152/jn.00093.2016 PMC4969396 27098024

[B8] DeFelipe J. Fariñas I. (1992). The pyramidal neuron of the cerebral cortex: morphological and chemical characteristics of the synaptic inputs. *Prog. Neurobiol.* 39 563–607. 10.1016/0301-0082(92)90015-7 1410442

[B9] Destexhe A. (2001). Simplified models of neocortical pyramidal cells. *Neurocomputing* 40 167–173. 10.1016/S0925-2312(01)00428-3

[B10] Elijah D. H. Samengo I. Montermurro M. A. (2015). Thalamic neuron models encode stimulus information by burst-size modulation. *Front. Comput. Neurosci.* 9:113 10.3389/fncom.2015.00113 PMC4585143 26441623

[B11] Feldman M. L. (1984). “Morphology of the neocortical pyramidal neuron,” in *Cerebral Cortex. Cellular Components of the Cerebral Cortex* Vol. 1 eds Peters A. Jones E. G. (New York, NY: Plenum Press), 123–200.

[B12] Fries P. (2005). A mechanism for cognitive dynamics: neuronal communication through neuronal coherence. *Trends Cogn. Sci.* 9 474–480. 10.1016/j.tics.2005.08.011 16150631

[B13] Fries P. (2015). Rhythms for cognition: communication through coherence. *Neuron* 88 220–235. 10.1016/j.neuron.2015.09.034 26447583 PMC4605134

[B14] Gray C. M. McCormick D. A. (1996). Chattering cells: superficial pyramidal neurons contributing to the generation of synchronous oscillations in the visual cortex. *Science* 274 109–113. 10.1126/science.274.5284.109 8810245

[B15] Gray H. (1918). *Anatomy of the Human Body.* Philadelphia, PA: Lea & Febiger 10.5962/bhl.title.20311

[B16] Häusser M. Spruston N. Stuart G. J. (2000). Diversity and dynamics of dendritic signaling. *Science* 290 739–744. 10.1126/science.290.5492.739 11052929

[B17] Horton J. C. Adams D. L. (2005). The cortical column: a structure without a function. *Philos. Trans. R. Soc. (Lon.). B Biol. Sci.* 360 837–862. 10.1098/rstb.2005.1623 15937015 PMC1569491

[B18] Hubel D. W. (1982). Cortical neurobiology: a slanted historical perspective. *Annu. Rev. Neurosci.* 5 363–370. 10.1146/annurev.ne.05.030182.002051 7041779

[B19] Innocenti G. M. Vercelli A. (2010). Dendritic bundles, minicolumns, columns, and cortical output units. *Front. Neuroanat.* 4:11 10.3389/neuro.05.011.2010 PMC2842100 20305751

[B20] Izhikevich E. M. Desai N. S. Walcott E. C. Hoppensteadt F. C. (2003). Bursts as a unit of neural information: selective communication via resonance. *Trends Neurosci.* 26 161–167. 10.1016/S0166-2236(03)00034-1 12591219

[B21] Jones E. G. (1998). Viewpoint: the core and matrix of thalamic organization. *Neuroscience* 85 331–345. 10.1016/S0306-4522(97)00581-2 9622234

[B22] Jones E. G. (2000). Microcolumns in the cerebral cortex. *Proc. Natl. Acad. Sci. (U.S.A.)* 97 5019–5021. 10.1073/pnas.97.10.5019 10805761 PMC33979

[B23] Jones E. G. (2002). Thalamic circuitry and thalamocortical synchrony. *Philos. Trans. R. Soc. Lond. B* 357 1659–1673. 10.1098/rstb.2002.1168 12626002 PMC1693077

[B24] Jones E. G. (2007). *The Thalamus*, 2nd Edn. Cambridge: Cambridge University Press.

[B25] Kasevich R. S. LaBerge D. (2011). Theory of electric resonance in the neocortical apical dendrite. *PLoS ONE* 6:e23412 10.1016/j.concog.2013.10.004 PMC3154468 21853129

[B26] Kepecs A. Lisman J. (2003). Information encoding and computation with spikes and bursts. *Comput. Neural. Syst.* 14 103–118. 10.1080/net.14.1.103.118 12613553

[B27] Keren N. Bar-Yehuda D. Korngreen A. (2009). Experimentally guided modeling of dendritic excitability in rat neocortical pyramidal neurons. *J. Physiol. (Lond.)* 587 1413–1437. 10.1113/jphysiol.2008.167130 19171651 PMC2678217

[B28] Keren N. Peled N. Korngreen A. (2005). Constraining compartmental models using multiple voltage recordings and genetic algorithms. *J. Neurophysiol.* 94 3730–3742. 10.1152/jn.00408.2005 16093338

[B29] LaBerge D. (2002). Attentional control: brief and prolonged. *Psychol. Res.* 66 220–233. 10.1007/s00426-002-0097-2 12466921

[B30] LaBerge D. (2005). Sustained attention and apical dendrite activity in recurrent circuits. *Brain Res. Rev.* 50 86–99. 10.1016/j.brainresrev.2005.04.004 15921761

[B31] LaBerge D. Auclair L. Sieroff E. (2000). Preparatory attention: experiment and theory. *Conscious. Cogn.* 9 396–434. 10.1006/ccog.1999.0429 10993667

[B32] LaBerge D. Kasevich R. S. (2013). The cognitive significance of resonating neurons in the cerebral cortex. *Cons. Cogn.* 22 1523–1550. 10.1016/j.concog.2013.10.004 24211318

[B33] Larkum M. (2013). A cellular mechanism for cortical associations: an organizing principle for the cerebral cortex. *Trends Neurosci.* 36 141–151. 10.1016/j.tins.2012.11.006 23273272

[B34] Lesica N. Stanley G. (2004). Encoding of natural scene movies by tonic and burst spikes in the lateral geniculate nucleus. *J. Neurosci.* 24 10731–10740. 10.1523/JNEUROSCI.3059-04.2004 15564591 PMC6730113

[B35] Llano D. A. Sherman S. M. (2009). Differences in intrinsic properties and local network connectivity of identified layer 5 and layer 6 adult mouse auditory corticothalamic neurons support a dual corticothalamic projection hypothesis. *Cereb.Cortex* 19 2810–2826. 10.1093/cercor/bhp050 19351905 PMC2774389

[B36] Marin-Padilla M. (1972). Structural abnormalities of the cerebral cortex in human chromosome aberrations: a Golgi study. *Brain Res.* 44 625–629. 10.1016/0006-8993(72)90324-1 4263073

[B37] Marlinski V. Beloozerova I. N. (2014). Burst firing of neurons in the thalamic reticular nucleus during locomotion. *J. Neurophysiol.* 112 181–192. 10.1152/jn.00366.2013 24740856 PMC4064390

[B38] Mease R. A. Metz M. Groh A. (2015). Cortical sensory responses are enhanced by the higher-order thalamus. *Cell* 14 208–215. 10.1016/j.celrep.2015.12.026 26748702

[B39] Mercer A. West D. C. Morris O. T. Kirchhecker S. Kerkhoff J. E. Thomson A. M. (2005). Excitatory connections made by presynaptic cortico-cortical pyramidal cells in layer 6 of the neocortex. *Cereb. Cortex* 15 1485–1496. 10.1093/cercor/bhi027 15647524

[B40] Mitchell A. S. Sherman S. M. Sommer M. A. Mair R. G. Vertes R. P. Chudasama Y. (2014). Advances in understanding mechanisms of thalamic relays in cognition and behavior. *J. Neurosci.* 34 15340–15346. 10.1523/JNEUROSCI.3289-14.2014 25392501 PMC4228136

[B41] Morel A. Gallay M. N. Baechler A. Wyss M. Gallay D. S. (2013). The human insula: architectonic organization and postmortem MRI registration. *Neuroscience* 236 117–135. 10.1016/j.neuroscience.2012.12.076 23340245

[B42] Mountcastle V. B. (1957). The columnar organization of the neocortex. *Brain* 120 701–722. 10.1093/brain/120.4.701 9153131

[B43] Mountcastle V. B. (1998). *The Cerebral Cortex.* Cambridge, MA: Harvard University Press.

[B44] Narayanan R. Johnston D. (2008). The h channel mediates location dependence and plasticity of intrinsic phase response in rat hippocampal neurons. *J. Neurosci* 28 5846–5860. 10.1523/JNEUROSCI.0835-08.2008 18509046 PMC2612942

[B45] Nieuwenhuys R. Voogd J. van Huizen C. (2008). *The Human Central Nervous System: A Synopsis and Atlas.* Berlin: Springer-Verlag, 564 10.1007/978-3-540-34686-9

[B46] Nolte J. (1988). *The Human Brain.* St. Louis, MI: C.W. Mosley.

[B47] Peters A. (1994). “The organization of the primary visual cortex in the macaque,” in *Cerebral Cortex*, eds Peters A. Rockland K. S. (New York, NY: Plenum Press).

[B48] Peters A. Sethares C. (1991). Organization of pyramidal neurons in area 17 of monkey visual cortex. *J. Comp. Neurol.* 306 1–23. 10.1002/cne.903060102 1710236

[B49] Phillips W. A. (2017). Cognitive functions of intracellular mechanisms for contextual amplification. *Brain Cogn.* 112 39–53. 10.1016/j.bandc.2015.09.005 26428863

[B50] Rakic P. (2008). Confusing cortical columns. *Proc. Natl. Acad. Sci. U.S.A.* 105 12099–12100. 10.1073/pnas.0807271105 18715998 PMC2527871

[B51] Rockel A. J. Horns R. W. Powell T. P. S. (1980). The basic uniformity in the structure of the neocortex. *Brain* 103 221–244. 10.1093/brain/103.2.221 6772266

[B52] Rockland K. S. (2010). Five points on columns. *Front. Neuroanat.* 4:22 10.3389/fnana.2010.00022 PMC2893004 20589097

[B53] Saalmann Y. B. Kastner S. (2011). Cognitive and perceptual functions of the visual thalamus. *Neuron* 71 209–223. 10.1016/j.neuron.2011.06.027 21791281 PMC3148184

[B54] Schiller J. Schiller Y. Stuart G. Sakmann B. (1997). Calcium action potentials restricted to distal apical dendrites of rat neocortical pyramidal neurons. *J. Physiol.* 505 605–616. 10.1111/j.1469-7793.1997.605ba.x 9457639 PMC1160039

[B55] Schwindt P. C. Crill W. E. (1995). Amplification of synaptic current by persistent sodium conductance in apical dendrite of neocortical neurons. *J. Neurophysiol.* 74 2220–2224. 8592214 10.1152/jn.1995.74.5.2220

[B56] Sherman S. M. (1996). Dual response modes in lateral geniculate neurons: mechanisms and functions. *Visual Neurosci.* 13 205–213. 10.1017/S0952523800007446 8737271

[B57] Sherman S. M. Guillery R. W. (1998). On the actions that one nerve cell can have on another: distinguishing “drivers” from ‘modulators’. *Proc. Natl. Acad. Sci. U.S.A.* 95 7121–7126. 10.1073/pnas.95.12.7121 9618549 PMC22761

[B58] Sherman S. M. Guillery R. W. (2011). Distinct functions for direct and trans-thalamic corticocortical connections. *J. Neurophysiol.* 106 1068–1077. 10.1152/jn.00429.2011 21676936

[B59] Shipp S. (2005). The importance of being agranular: a comparative account of visual and motor cortex. *Philos. Trans. R. Soc. Lond. B Biol. Sci.* 360 797–814. 10.1098/rstb.2005.1630 15937013 PMC1569485

[B60] Silva L. R. Amitai Y. Connors B. W. (1991). Intrinsic oscillations of neocortex generated by layer 5 pyramidal neurons. *Science* 25 432–435. 10.1126/science.1824881 1824881

[B61] Spruston N. (2008). Pyramidal neurons: dendritic structure and synaptic integration. *Nat. Rev. Neurosci.* 9 206–221. 10.1038/nrn2286 18270515

[B62] Thomson A. M. (2010). Neocortical layer 6 a review. *Front. Neuroanat.* 4:13 10.3389/fnana.2010.00013 PMC2885865 20556241

[B63] Valverde F. (1967). Apical dendritic spines of the visual cortex and light deprivation in the mouse. *Exp. Brain Res.* 3 337–352. 10.1007/BF00237559 6031165

[B64] Valverde F. (1986). Intrinsic neocortical organization: Some comparative aspects. *Neuroscience* 18 1–23. 10.1016/0306-4522(86)90174-0 3090475

[B65] Vercelli A. E. Garbosa D. Curtetti R. Innocenti G. M. (2004). Somatodendritic minicolumns of output neurons in the rat visual cortex. *Eur. J. Neurosci.* 20 495–502. 10.1111/j.1460-9568.2004.03483 15233758

[B66] Wallis J. D. (2012). Cross-species studies of orbitofrontal cortex and value-based decision-making. *Nat. Neurosci.* 15 13–19. 10.1038/nn.2956 PMC3549638 22101646

[B67] Wang S.-J. (2010). Neurophysiological and computational principles of cortical rhythms in cognition. *Physiol. Rev.* 90 1195–1268. 10.1152/physrev.00035.2008 20664082 PMC2923921

[B68] Wiser A. K. Callaway E. M. (1996). Contributions to individual layer 6 pyramidal neurons to local circuit in macaque primary visual cortex. *J. Neurosci.* 16 2724–2739. 8786448 10.1523/JNEUROSCI.16-08-02724.1996 PMC6578755

[B69] Womelsdorf T. Everling S. (2015). Long-range attention networks: circuit motifs underlying endogenously controlled stimulus selection. *Trends Neurosci.* 38 682–700. 10.1016/j.tins.2015.08.009 26549883

[B70] Womelsdorf T. Salva A. Everling S. Valiante T. A. (2014). Burst firing synchronizes prefrontal and anterior cingulate cortex during attentional control. *Curr. Biol.* 24 2613–2621. 10.1016/j.cub.2014.09.046 25308081

[B71] Womelsdorf T. Schoffelen J.-M. Oostenveld R. Singer W. Desimone R. Engel A. K. (2007). Modulation of neuronal interactions through neuronal synchronization. *Science* 316 1609–1612. 10.1126/science.1139597 17569862

[B72] Zhu J. J. (2000). Maturation of layer 5 neocortical pyramidal neurons: amplifying salient layer 1 and layer 4 inputs by Ca^2+^ action potentials in adult rat tuft dendrites. *J. Physiol.* 526 571–587. 10.1111/j.1469-7793.2000.00571.x 10922009 PMC2270034

[B73] Zikopoulos B. Barbas H. (2007). Parallel driving and modulatory pathways link the prefrontal cortex and thalamus. *PLoS ONE* 2:e848 10.1371/journal.pone.0000848 PMC1952177 17786219

